# Membrane therapy using DHA suppresses epidermal growth factor receptor signaling by disrupting nanocluster formation

**DOI:** 10.1016/j.jlr.2021.100026

**Published:** 2021-01-27

**Authors:** Natividad R. Fuentes, Mohamed Mlih, Xiaoli Wang, Gabriella Webster, Sergio Cortes-Acosta, Michael L. Salinas, Ian R. Corbin, Jason Karpac, Robert S. Chapkin

**Affiliations:** 1 Program in Integrative Nutrition and Complex Diseases, Texas A&M University, College Station, TX, USA; 2 Department of Nutrition, Texas A&M University, College Station, TX, USA; 3 Interdisciplinary Faculty of Toxicology, Texas A&M University, College Station, TX, USA; 4 Department of Molecular and Cellular Medicine, College of Medicine, Texas A&M Health Science Center, Bryan, TX, USA; 5 Advanced Imaging Research Center, University of Texas Southwestern Medical Center, Dallas, TX, USA; 6 Center for Translational Environmental Health Research, Texas A&M University, College Station, TX, USA

**Keywords:** Cancer, Cholesterol, Membranes/Fluidity, Omega-3 fatty acids, Receptors/Plasma membrane, Super-resolution microscopy, DHA, docosahexaenoic acid, DPBS, Dulbecco's phosphate buffered saline, EGF, epidermal growth factor, EGFR, epidermal growth factor receptor, FIPI, 5-fluoro-2-indolyl des-chlorohalopemide, FLIM, fluorescence lifetime imaging, FRET, fluorescence resonance energy transfer, IMCE, immortalized murine colonic epithelial, ISC, intestinal stem cell, LA, linoleic acid, LSD, least significant difference, MβCD, methyl-beta-cyclodextrin, OA, oleic acid, PA, phosphatidic acid, PAO, phenylarsine oxide, PIP2, phosphatidylinositol-4,5-bisphosphate, STED, stimulated emission depletion, STORM, stochastic optical reconstruction microscopy, YAMC, young adult mouse colonic

## Abstract

Epidermal growth factor receptor (EGFR) signaling drives the formation of many types of cancer, including colon cancer. Docosahexaenoic acid (DHA, 22∶6^Δ4,7,10,13,16,19^), a chemoprotective long-chain n-3 polyunsaturated fatty acid suppresses EGFR signaling. However, the mechanism underlying this phenotype remains unclear. Therefore, we used super-resolution microscopy techniques to investigate the mechanistic link between EGFR function and DHA-induced alterations to plasma membrane nanodomains. Using isogenic in vitro (YAMC and IMCE mouse colonic cell lines) and in vivo (*Drosophila*, wild type and *Fat-1* mice) models, cellular DHA enrichment via therapeutic nanoparticle delivery, endogenous synthesis, or dietary supplementation reduced EGFR-mediated cell proliferation and downstream Ras/ERK signaling. Phospholipid incorporation of DHA reduced membrane rigidity and the size of EGFR nanoclusters. Similarly, pharmacological reduction of plasma membrane phosphatidic acid (PA), phosphatidylinositol-4,5-bisphosphate (PIP_2_) or cholesterol was associated with a decrease in EGFR nanocluster size. Furthermore, in DHA-treated cells only the addition of cholesterol, unlike PA or PIP_2_, restored EGFR nanoscale clustering. These findings reveal that DHA reduces EGFR signaling in part by reshaping EGFR proteolipid nanodomains, supporting the feasibility of using membrane therapy, i.e., dietary/drug-related strategies to target plasma membrane organization, to reduce EGFR signaling and cancer risk.

## Summary

Cellular membrane phospholipid enrichment of DHA suppresses EGFR-mediated phenotypes by reducing EGFR nanocluster formation across a variety of in vitro and in vivo models.

The epidermal growth factor receptor (EGFR) is a transmembrane receptor tyrosine kinase that is mutated or overexpressed in many cancerous tissues, including the colon (1). EGFR signaling mediates many cellular processes involved in epithelial tissue homeostasis (2). Inhibition of EGFR signaling has been shown to reduce uncontrolled cell growth (3), and EGFR targeted pharmaceuticals are used in the treatment of colorectal cancer (4). However, undesirable side effects (5) and acquired resistance (6, 7) to these therapeutics highlight the need for the development of alternative strategies.

Mounting experimental, epidemiological, and clinical evidence suggest that consumption of n-3 polyunsaturated fatty acids (PUFAs), including docosahexaenoic acid (DHA, 22∶6^Δ4,7,10,13,16,19^), is protective against colon tumorigenesis (8, 9, 10, 11). Furthermore, preclinical evidence support the role of DHA as an adjuvant therapeutic for colon cancer (12, 13, 14, 15, 16, 17, 18). Of particular interest is the paradoxical ability of DHA to increase EGFR phosphorylation yet attenuate epidermal growth factor (EGF)-mediated Ras activation and subsequent ERK phosphorylation (15, 16). However, the underlying cause for this disruption is unclear, highlighting the need to elucidate the molecular mechanism by which DHA suppresses EGFR signaling.

EGFR signaling is initiated by the binding of ligands, such as EGF, which induces conformational changes that support receptor dimerization, autophosphorylation, the subsequent recruitment of signaling adaptors Grb2 and Sos1, downstream effectors such Ras (19), and ultimately the activation of ERK (20). However, it was recently established that EGFR dimerization, phosphorylation, and recruitment of Grb2 and Sos1 is not sufficient for Ras activation (21). A prevailing hypothesis argues that EGFR signaling is influenced by the formation of nanoscale clusters (referred to as nanoclusters) in the plasma membrane, which are maintained by protein-lipid interactions between EGFR, phosphatidic acid (PA), phosphatidylinositol-4,5-bisphosphate (PIP_2_), and cholesterol (22, 23, 24, 25). The clinical relevance of EGFR nanocluster formation is supported by observations indicating that the number and size of EGFR nanoclusters is increased in epithelial cancers (23), providing rationale for the targeted disruption of EGFR nanocluster formation as a novel therapy. Thus, the development of membrane therapy could provide a complementary tool against EGFR-driven cancer.

Herein, we report the reduction of EGFR nanocluster formation as an underlying cause for DHA-mediated EGFR signal attenuation. By incorporation into membrane phospholipids, DHA reduces the rigidity of the plasma membrane, interfering with EGFR-cholesterol interactions, leading to the collective reduction in EGFR cluster formation, downstream Ras activation, and ERK signaling. Together, these results support the feasibility of utilizing DHA in the modulation of EGFR nanoscale spatial organization and signaling.

## Materials and methods

### Cell culture

Conditionally immortalized young adult mouse colonic (YAMC) epithelial (RRID: CVCL_6E40) and immortalized murine colonic epithelial (IMCE) cells were originally obtained from R.H. Whitehead, Ludwig Cancer Institute (Melbourne, Australia). YAMC (passages 12–20) and IMCE (passages 19–26) cells were cultured under permissive conditions, 33°C, and 5% CO_2_ in Roswell Park Memorial Institute (RPMI) 1640 medium, no glutamine (Gibco, 21870076) supplemented with 5% fetal bovine serum (FBS; Hyclone, SH300084.03), 2 mM GlutaMAX (Gibco, 35050061), 5 μg/mL insulin, 5 μg/mL transferrin, 5 ng/mL selenious acid (Corning, 354351), and 5 IU/mL of murine interferon-g (Roche, 11276905001). SW48 cells (RRID:CVCL_1724) were obtained (10/08/14) from Horizon Discovery (Cambridge, United Kingdom), where they were authenticated by gDNA and cDNA genotyping. SW48 cells (passages 8–13) were maintained at 37°C and 5% CO_2_ in McCoy's 5A medium supplemented with 10% FBS. YAMC cells were authenticated (07/24/15) by STR profiling (CellCheck Plus) by IDEXX BioResearch (Westbrook, ME). All cell lines used tested negative for mycoplasma bacteria (05/09/18) as assessed by a Universal Mycoplasma Detection Kit (ATCC, 30-1012K). Select cultures were treated for 24 or 72 h with 50 μM fatty acid [oleic acid (OA, 18:1n9), linoleic acid (LA, 18:2n6), arachidonic acid (AA, 20:4n6), or DHA (22:6n3); Nu-Chek Prep, Inc., Elysian, MN] complexed with fatty acid-free bovine serum albumin (BSA) or low density lipoproteins (LDLs). Select cultures were treated for 24 h with LDL-OA or LDL-DHA. Incorporation of unesterified DHA (Nu-Chek Prep, Inc., Elysian, MN) into LDL was performed by the reconstitution method, as described in our previous publication (26). BSA-DHA, LDL-OA, and LDL-DHA nanoparticle lipid composition is described in supplemental Table S1.

### EGF-dependent colonoid growth assay

Ninety IMCE cells were seeded in 3 mL Matrigel per well in a 96-well plate using RPMI supplemented with 10% FBS and IFN-γ at 33°C. On the following day medium containing EGF (25 ng/mL) was added to allow colonies to establish. After 48 h, medium containing EGF and the indicated treatments (50 μM) was added. Organoids were grown for 10 days while changing media every 3–4 days. Cells were imaged with a 2× objective on a Keyence microscope (BZ X-710 fluorescent microscope, RRID:SCR_017202). Keyence analyzer software (BZ Analyzer software, RRID:SCR_017205) was used to generate a full focused image from a Z-stacks of 30 planes at 20 μm steps. For analysis, full focused images were opened in National Institutes of Health ImageJ software (ImageJ, RRID:SCR_003070; Fiji, RRID:SCR_002285), and a custom macro was used to quantify colonoid surface coverage.

### Spatiotemporal Ras biosensor fluorescence resonance energy transfer imaging

YAMC cells were untreated or treated with indicated fatty acids (50 μM) for 24 h, then transfected with plasmid encoding the KRas-Raichu biosensor. Cells were subsequently incubated an additional 48 h and starved in Phenol-free RPMI (0.5%, FBS), 1% Glutamax, 1% Pen/Strep, with IFN-γ, no insulin-transferrin-selenium, for 4 h before stimulation with EGF (25 ng/mL), and images were taken at 40× magnification with 4 × 4 binning every 2 min. The fluorescence resonance energy transfer (FRET)/cyan fluorescent protein ratio of each cell was normalized by dividing by the averaged FRET/cyan fluorescent protein value before stimulation, as previously described (27).

### Drosophila genetics, stocks, and culture

The following strains were obtained from the Bloomington *Drosophila* Stock Center: w1118, EGFR (9535), UAS-he-EGFR-GFP (58415) and tub-Gal80ts (DGGR Cat# 130454, RRID:DGGR_130454). esg-Gal4 was kindly provided by Dr Shigeo Hayashi, Riken Center for Developmental Biology. All flies were reared on standard yeast- and cornmeal-based diet at 25°C and 65% humidity on a 12 h light/dark cycle, unless otherwise indicated. The standard laboratory diet (cornmeal based) was made with the following protocol: 14 g agar/165.4 g malt extract/41.4 g dry yeast/78.2 g cornmeal/4.7 mL propionic acid/3 g methyl 4-hydroxybenzoate/1.5 liters water. All analyses were exclusively done in female flies because of sex-specific differences in midgut regeneration.

### Mouse genetics, husbandry, and diet

All animal experiments were approved and conducted in strict accordance with the Texas A&M University Institutional Animal Care and Use Committee and conformed to National Institutes of Health guidelines. *Fat-1* transgenic mice were generated and backcrossed onto a C57BL/6 background as previously described (28). The colony of *fat-1* mice used for this study was generated by breeding heterozygous mice. The genotype and phenotype of offspring of each animal were characterized using isolated DNA and total lipids from mice tail clips (29).

Mice were housed in cages in a temperature- and humidity-controlled animal facility with a 12 h light/dark cycle and fed a 10% safflower oil diet (Research Diets) ad libitum. The diet contained (g/100 g diet) 40 sucrose, 20 casein, 15 corn starch, 0.3 dl-methionine, 3.5 AIN 76A salt mix, 1.0 AIN 76A mineral mix, 0.2 choline chloride, 5 fiber (cellulose), and 10 safflower oil.

### Super-resolution microscopy labeling

For labeling cells for nanocluster analysis using stochastic optical reconstruction microscopy (STORM), YAMC cells were seeded in cell imaging 8 chamber coverglass slides (Cellvis, C8-1.5H-N) and allowed to attach for 24 h. Cells were subsequently treated with control RPMI media (5% FBS), LDL-OA (50 μM), or LDL-DHA (50 μM) for 24 h (26). After 24 h, cells were fixed with prewarmed (37°C) 4% cytoskeleton stabilizing buffer-paraformaldehyde for 15 min at room temperature (30, 31). After rinsing with Dulbecco's phosphate buffered saline (DPBS), cells were blocked with 5% BSA-DPBS for 30 min and subsequently incubated with 0.5 μg/mL EGF-Alexa647 (ThermoFisher, E35351) in 1% BSA-DPBS for 30 min at room temperature. Cells were rinsed with 1% BSA-DPBS twice and then with DPBS twice prior to imaging.

For labeling primary murine colonic cells for nanocluster analysis using STORM, colonic crypts were isolated as previously described (32). Isolated crypts were incubated in TrypLE™ Select Enzyme (10×) (ThermoFisher, A1217701) for 30 min at 37°C, pipetted up and down every 5–10 min, and then passed through a 40 μm cell strainer. Cells were suspended in live cell imaging solution (ThermoFisher, A14291DJ) and seeded in cell imaging eight chamber coverglass slides (Cellvis, C8-1.5H-N) coated with poly-d-lysine (ThermoFisher, A3890401) and allowed to attach for 30 min on ice before fixing with 4% cytoskeleton stabilizing buffer-paraformaldehyde (ice cold) for 15 min on ice (30). After rinsing with DPBS, cells were blocked with 5% BSA-DPBS for 30 min. Cells were subsequently incubated with 0.5 μg/mL EGF-Alexa647 (ThermoFisher, E35351) in 1% BSA-DPBS for 30 min at room temperature. Cells were rinsed with 1% BSA-DPBS twice and then with DPBS twice prior to imaging.

For labeling *Drosophila* gut esgG4 cells for nanocluster analysis using STORM, intact fly guts were fixed at room temperature for 20 min in 100 mM glutamic acid, 25 mM KCl, 20 mM MgSO_4_, 4 mM sodium phosphate, 1 mM MgCl_2_, and 4% formaldehyde. After rinsing with DPBS, guts were blocked with 5% BSA-DPBS for 30 min. Subsequently, guts were incubated with 2.5 μg/mL cetuximab-Alexa594 (R&D Systems, FAB9577T) overnight at 4°C. Guts were rinsed with DPBS 1% BSA twice and then with DPBS twice more prior to mounting in Mowiol medium.

### Direct stochastic optical reconstruction microscopy and stimulated emission depletion super-resolution microscopy imaging and nanocluster analysis

For details regarding STORM and stimulated emission depletion (STED) super-resolution imaging please see supplemental methods.

### Membrane order measurement via image-based flow cytometry

Cells were stained with Di-4-ANEPPDHQ (Invitrogen, D36802) for membrane order determination as previously described (33, 34, 35, 36). In brief, cells were stained with 1 μM Di4 and imaged via image-based flow cytometry, Amnis FlowSight (35, 36). Laser light at 488 nm was used to excite Di4 and emission wavelengths and subsequently collected in two preset channels representing ordered (O: 480–560 nm) and disordered (D: 640–745 nm). Generalized Polarization (GP) was calculated using the equation below: GP = (Intensity(O) − G × Intensity(D))/(Intensity(O) + G × Intensity(D)). Because there is no way to acquire a calibration image, the G factor was omitted and GP was calculated as stated above using Amnis IDEAS software.

### Statistical analysis

Statistical significance between treatments as indicated by uncommon letters (*P* < 0.01) was analyzed using one-way ANOVA and uncorrected Fisher's least significant difference (LSD) tests. All analyses were conducted using Prism statistical software (GraphPad Software, Inc.).

## Results

### DHA attenuates EGFR-mediated phenotypes

**Fig. 1:**
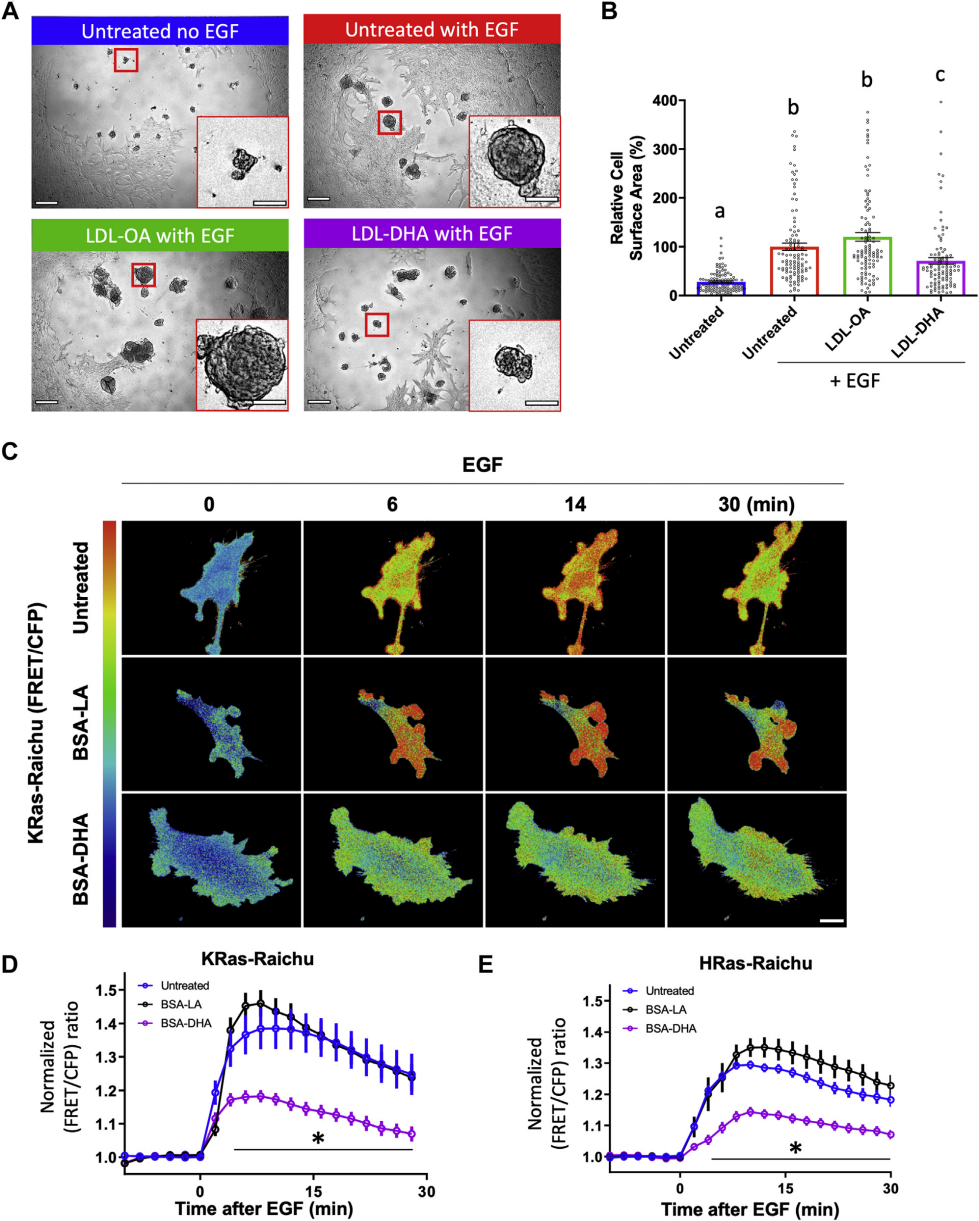
Exogenous supplementation with DHA reduces EGFR-dependent proliferation and downstream signaling in vitro. A: Representative images of colonoids grown with indicated treatments. Scale bar, 300 and 100 μm. B: Quantification of colonoid surface area after 10 days of treatment. Data represent mean ± SE. Number of organoids examined per treatment, Untreated No EGF = 112, Untreated + EGF = 109, LDL-OA + EGF = 109, LDL-DHA + EGF = 112, from eight wells per group from two independent experiments. Statistical significance between treatments as indicated by uncommon letters (*P* < 0.05) was examined using one-way ANOVA and uncorrected Fisher's LSD tests. C: Spatiotemporal activation of Ras was determined by monitoring activation of FRET biosensors targeted to (D) K- or (E) H-Ras domains. C: Representative intensity-modulated images of KRas-Raichu–expressing cells at various time points following EGF stimulation. Scale bar, 20 μm. D: Data represent mean ± SE, FRET ratio for each cell. Number of cells examined per treatment, Untreated = 8, BSA-LA = 11, and LDL-DHA = 8. All points after 4 min are statistically significant (*P* < 0.05) between BSA-DHA and untreated (control) as indicated by bar and (∗). E: Data represent mean ± SE, FRET ratio for each cell. Number of cells examined per treatment, Untreated = 26, BSA-LA = 10, and LDL-DHA = 22. All points after 4 min are statistically significant (*P* < 0.05) between BSA-DHA and untreated (control) as indicated by bar and (∗).

To assess the ability of DHA to modulate EGFR-driven proliferation, we utilized a 3D model of EGF-dependent growth, i.e., the (IMCE) cell line, which is a nontransformed normal colonic epithelial cell line, carrying one mutated APC allele, which is conditionally immortalized by expression of a temperature-sensitive simian virus 40 (SV40) large T antigen (37). IMCE cells do not form colonies in soft agar without the addition of an oncogenic Ras mutation (37). Since oncogenic Ras is downstream of EGFR, we hypothesized that the addition of exogenous EGF would allow IMCE cells to form 3D colonies, i.e., colonoids, in Matrigel™. Supplementing media with EGF (25 ng/mL) induced the formation of IMCE colonoids (supplemental Fig. S1A, B). Colonoid size was EGF dependent, since EGF withdrawal produced smaller colonoids as determined by surface area assessment (supplemental Fig. S1C, D). To determine the effect of DHA supplementation on EGFR-dependent growth, we utilized a DHA-based therapeutic consisting of DHA free fatty acid inserted into human LDL particles (26). Each LDL nanoparticle is devoid of cholesterol, contains approximately 1,100 unesterified DHA fatty acid molecules (LDL-DHA), and is approximately 22 nm in diameter (38). These nanoparticles have been shown to preferentially and efficiently target liver cancer versus noncancer cells in vitro (26, 39) and reduce proliferation of xenografts of hepatocellular carcinoma (HepG2) in vivo. LDL-DHA treatment reduced colonoid size as compared with untreated and monounsaturated fatty acid (OA) controls (Fig. 1A, B). This is consistent with our previous studies demonstrating the EGFR dependency of DHA with regard to cell proliferation by comparing its effects in wild type versus EGFR null YAMC cells (15). Next, we assessed the impact of DHA on EGF-mediated spatiotemporal activation of K- and H-Ras. Nontransformed conditionally immortalized YAMC cells, which contain a temperature-sensitive mutation of the SV40 large T antigen gene, were treated with fatty acid complexed with BSA and subsequently transfected with plasmids encoding K- or H-Ras-Raichu FRET-based biosensors (40, 41). DHA treatment attenuated temporal activation of K- and H-Ras biosensors compared with untreated and PUFA (e.g., LA)-treated controls (Fig. 1C–E). Collectively, these findings demonstrate that in vitro delivery of DHA functionally impairs EGFR-dependent signaling in colonic cells, consistent with previous observations (15, 16).

**Fig. 2:**
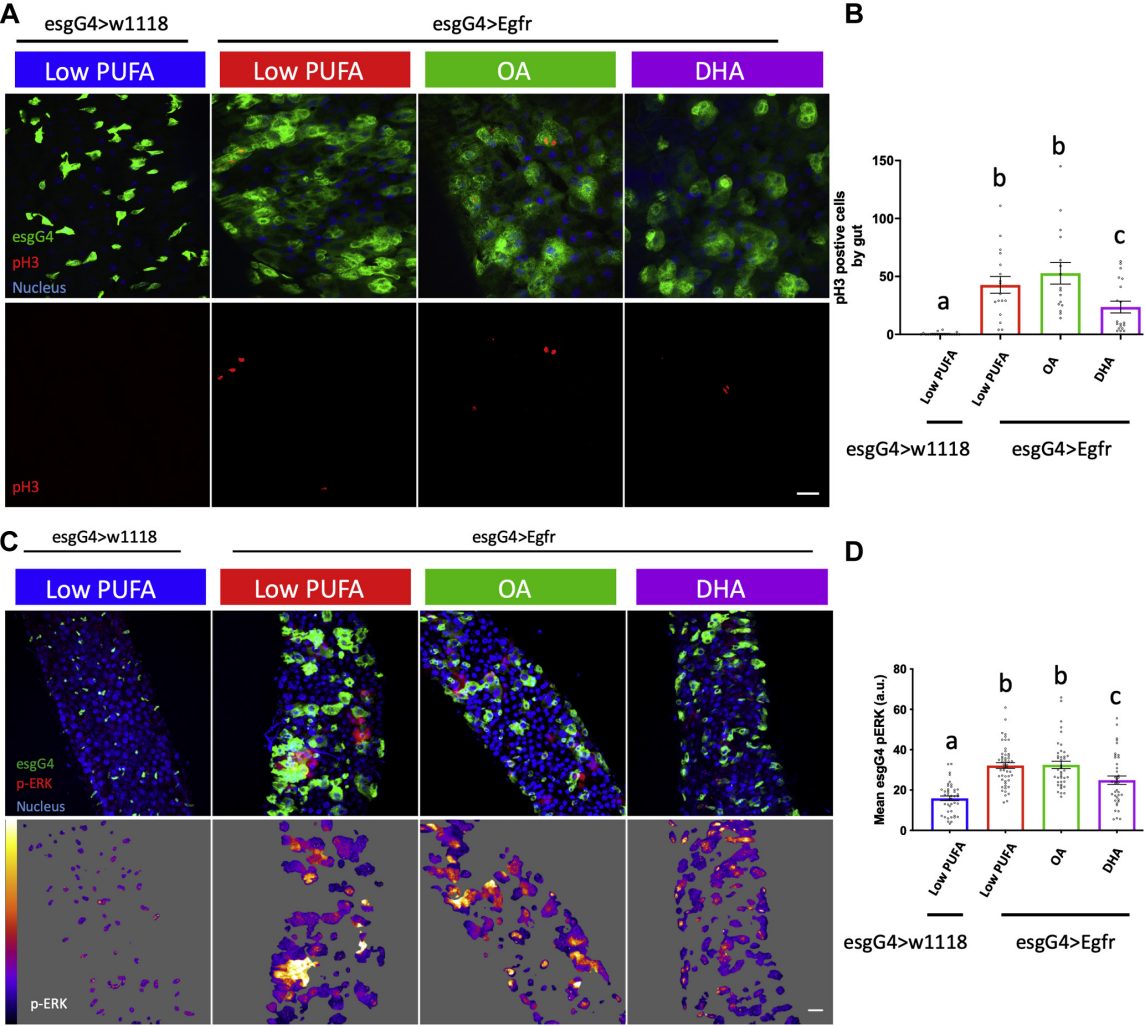
Dietary DHA reduces EGFR overexpression–driven proliferation and ERK activation. Adult *Drosophila* were placed on control (PUFA Free) or OA- or DHA-enriched diets for 5 days at 18°C (permissive temperature) before switching to 29°C for 2 days to induce EGFR overexpression in gut esgG4 cells. A: Representative merged and pH3 images. Scale bar, 20 μm. B: Quantitative analysis of proliferation as assessed by pH3 at 48 h post EGFR induction. Data represent mean ± SE from 16–21 guts from three independent experiments. C: Representative merged maximum image projection and masked esgG4 stem cell pERK. Scale bar, 20 μm. Quantitative analysis of (D) mean pERK in esgG4 cells per field of view (FOV) from flies fed the experimental diets. Data represent mean ± SE from 39–51 FOV from 30 guts from three independent experiments. Statistical significance between treatments as indicated by uncommon letters (B, *P* < 0.05; D, *P* < 0.01) was determined using one-way ANOVA and uncorrected Fisher's LSD tests.

In complementary experiments, we determined whether dietary DHA modulates EGFR-mediated phenotypes in vivo. For this purpose, the *Drosophila* intestinal (midgut) epithelium model was utilized. The presence of somatic intestinal stem cells (ISCs) within the fly midgut allows for the use of a wide range of genetic tools to assay signaling events that govern proliferative homeostasis in vivo (42). This barrier epithelium, with functional and morphological similarities to the mammalian small intestine and mouse airway epithelia (42), contains ISCs that can asymmetrically divide, forming an enteroblast that directly differentiates into functional enterocytes. Thus, the *Drosophila* ISC lineage provides an excellent model to study signaling mechanisms regulating stem cell maintenance and dysfunction, including EGFR-mediated proliferative signaling (2, 43). To extend our findings in IMCE cells, we assessed the effects of a DHA-enriched diet on EGFR-dependent proliferation and signaling in *Drosophila* using transgenic flies that overexpress dEGFR specifically within somatic ISC and enteroblasts (using the EsgGal4, GFP driver). Flies were fed a low-PUFA diet (44) with or without the addition of 0.5% w/w DHA or OA, a control monounsaturated fatty acid. As expected, overexpression of dEGFR targeted to ISCs in the adult fly increased stem cell proliferation and ERK signaling (Fig. 2A–D). Flies fed DHA verses low PUFA (basal) or OA control diets exhibited a reduction in stem cell proliferation (Fig. 2A, B) and ERK activation (Fig. 2C, D). Collectively, these data demonstrate that DHA suppresses EGFR-dependent proliferative phenotypes in vivo.

### Plasma membrane biophysical properties are altered by DHA

**Fig. 3:**
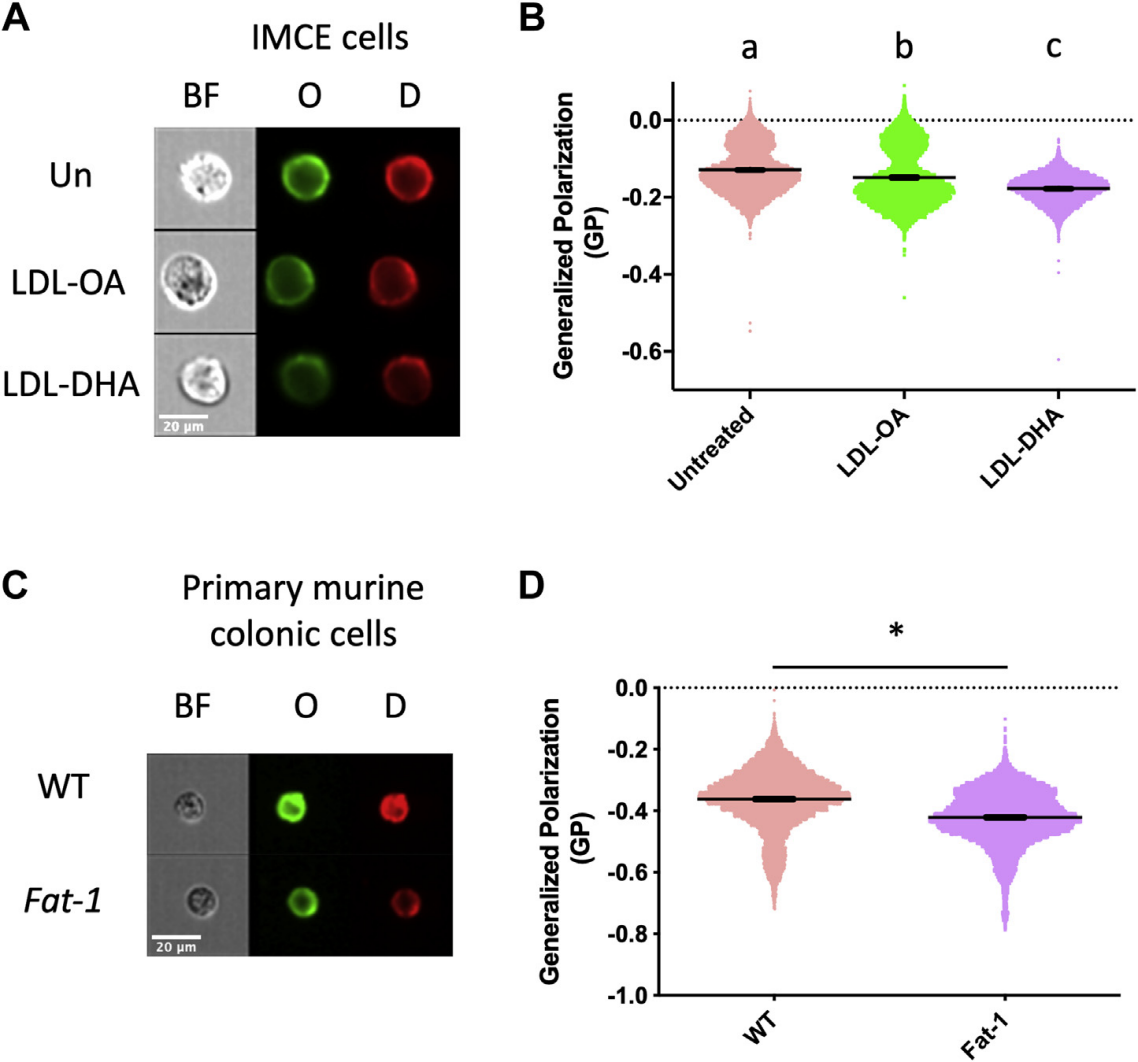
DHA reduces plasma membrane rigidity. IMCE cells were incubated with the indicated treatments (50 μM) for 24 h before labeling with Di-4-ANEPPDHQ, followed by assessment of membrane order using imaged based flow cytometry. A: Representative images and (B) quantitative analysis of membrane order in IMCE cells. Data represent mean ± SE from individual cells from untreated (13,240), LDL-OA (12,202), and LDL-DHA (14,674), from three independent experiments. Statistical significance between treatments as indicated by uncommon letters (*P* < 0.0001) was examined using one-way ANOVA and uncorrected Fisher's LSD tests. Single cells from wild-type or *Fat-1* mice were labeled with Di-4-ANEPPDHQ and imaged via imaging flow cytometry. C: Representative images and (D) quantitative analysis of membrane order in isolated primary murine colonic cells. Data represent mean ± SE from individual cells from WT (12,655) and *Fat-1* (16,675) from 3 and 4 mice, respectively. Statistical significance between groups (∗*P* < 0.0001) was examined using an unpaired *t*-tests.

We next investigated the mechanism by which DHA impairs EGFR function. With 22 carbons and six double bonds, DHA is the longest and most unsaturated fatty acid commonly found in human membranes. When incorporated into membrane phospholipids, including caveolae and lipid raft domains (45, 46), the unique biophysical characteristics of DHA can influence membrane structure (33, 47, 48). Since the biophysical properties of the plasma membrane can modulate plasma membrane receptor function (36) and downstream cellular signaling (49), we initially explored the effects of LDL-DHA on plasma membrane rigidity. For this purpose, IMCE cells were labeled with a polarity-sensitive dye, Di-4-ANEPPDHQ (Di4) (34, 35), and individually imaged (Fig. 3A). LDL-DHA treatment reduced plasma membrane rigidity (Fig. 3B). To determine if DHA modulates plasma membrane biophysical properties in vivo, colonocytes were isolated from *Fat-1* transgenic mice, a genetic model in which phospholipids are enriched via de novo synthesis of DHA (28, 29). Of note, plasma membrane rigidity was also reduced in primary colonic cells isolated from *Fat-1* mice versus wild-type controls (Fig. 3C, D). Overall, these findings underscore the ability of DHA to modulate plasma membrane biophysical properties by reducing plasma membrane rigidity.

### DHA attenuates EGFR nanocluster formation

**Fig. 4:**
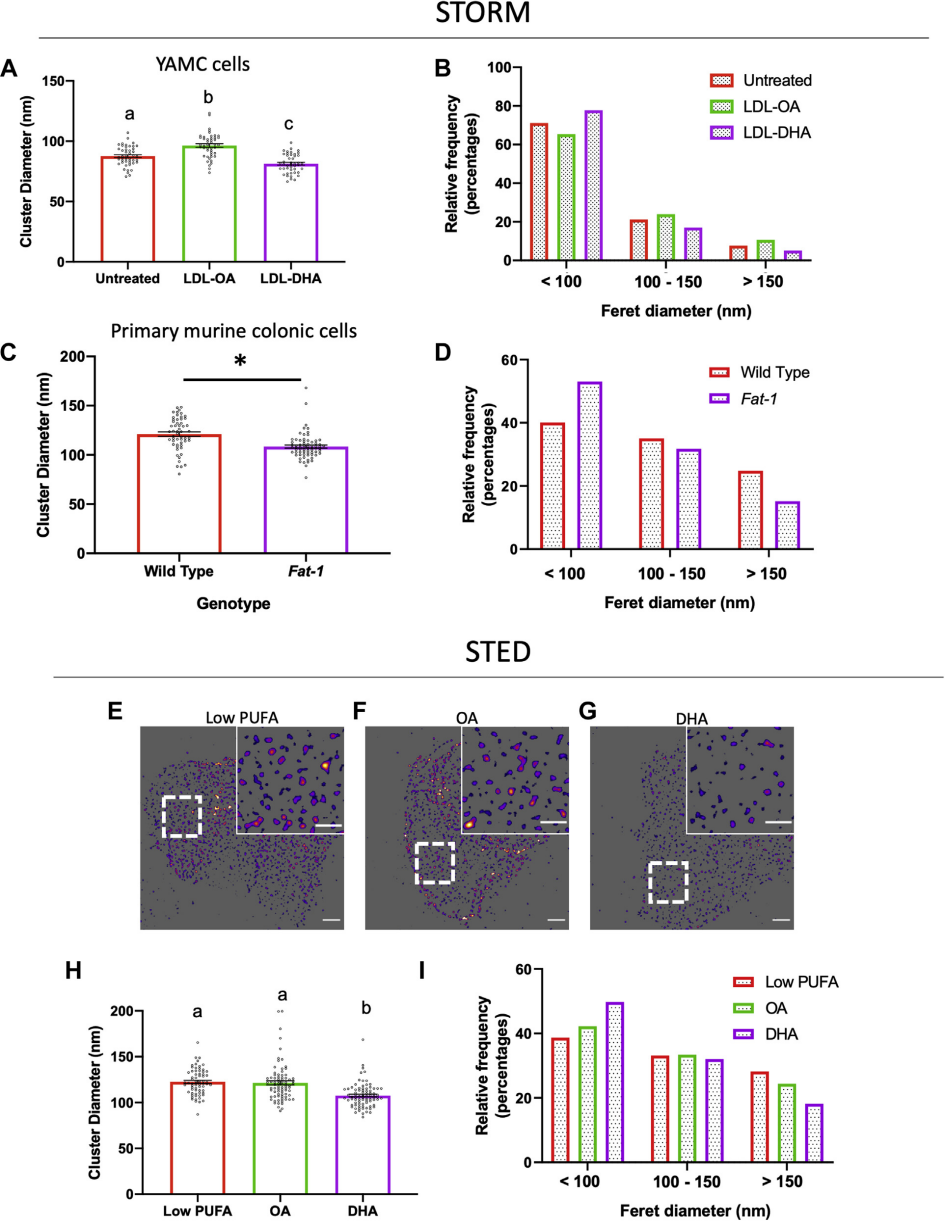
DHA reduces EGFR nanoclustering. YAMC cells were incubated with the indicated treatments (50 μM) for 24 h prior to fixation and subsequent labeling with EGF-Alexa647 for STORM imaging. A: Quantitative analysis of EGFR cluster diameter and (B) relative cluster size in YAMC cells. Data are presented as (A) mean ± SE of average EGFR cluster diameter per FOV and (B) individual cluster distribution. Number of FOVs examined per treatment, untreated = 46, LDL-OA = 46, LDL-DHA = 46, and individual clusters, untreated = 4,824, LDL-OA = 8,305, LDL-DHA = 2,921, from four wells per group from two independent experiments. C: Quantitative analysis of EGFR cluster diameter and (D) relative cluster size in isolated primary murine colonic cells. Data are presented as (C) mean ± SE of average EGFR cluster diameter per FOV, and (D) individual cluster size distribution. Number of FOVs examined per group, wild type = 55 and *Fat-1* = 69, and individual clusters, wild type = 4,742 and *Fat-1* = 3,872, from 3 or 4 mice, respectively. Adult *Drosophila* were placed on control (PUFA Free) or OA- or DHA-enriched diets for 5 days at 18°C (permissive temperature) before switching to 29°C for 2 days to induce chimeric human EGFR expression in gut esgG4 cells. E–G: Representative processed STED images from indicated diet. Scale bar, 1 μm and 500 nm. H: Quantitative analysis of EGFR cluster diameter and (I) relative frequency in *Drosophila* gut esgG4 cells. Data are presented as (H) mean ± SE of average EGFR cluster diameter per FOV, and (I) individual cluster size distribution. Number of FOVs examined per group, Low PUFA = 68, OA = 80, and DHA = 82, and individual clusters, Low PUFA = 51,442, OA = 56,471, and DHA = 35,103, from three independent experiments. Unless otherwise indicated, statistical significance between groups as indicated by uncommon letters (*P* < 0.001) was analyzed using one-way ANOVA and uncorrected Fisher's LSD tests.

The observed effect of DHA on plasma membrane biophysical properties is relevant since EGF stimulation can induce the formation of cholesterol-enriched rigid plasma membrane domains, i.e., lipid raft components (50, 51, 52). Since plasma membrane rigidity is influenced by many factors including lipid saturation (33, 53, 54), cholesterol content (55, 56), and cytoskeletal interactions (57), its modulation may not be correlated to protein clustering in the plasma membrane (58). Therefore, we used super-resolution microscopy to determine if DHA incorporation into membrane phospholipids reduces EGFR cluster size. We first validated the functionality and specificity of fluorophore complexed EGF. EGF-488 showed strong internalization at 37°C but not 4°C (supplemental Fig. S2A, B) and was specific for EGFR, e.g., it failed to label EGFR-negative cells (supplemental Fig. S2C). In subsequent experiments, EGFR clustering in YAMC cells was assessed by STORM (supplemental Fig. S3). EGFR nanocluster size was reduced by LDL-DHA (Fig. 4A, B; supplemental Fig. S4A–C) and BSA-DHA (supplemental Fig. S5) treatment as compared with LDL-OA and untreated controls. Of note, EGFR clustering was reduced by LDL-DHA and BSA-DHA treatment in SW48 colorectal adenocarcinoma cells (supplemental Fig. S6), which express high levels of EGFR (59) containing a G719S mutation, which is constitutively active and resistant to kinase inhibitors (60, 61). Because delivery of DHA by LDL nanoparticles is pharmacological in nature, we verified that LDL-DHA treatment enhanced the DHA composition of cellular phospholipids to a similar degree as compared with the physiological method of DHA delivery using BSA-DHA (supplemental Table S2). Furthermore, isolated colonocytes from *Fat-1* (genetically enriched with DHA) versus wild-type litter-mate control mice contained smaller EGFR clusters (Fig. 4C, D; supplemental Fig. S7A, B). Therefore, we investigated the in vivo effects of dietary DHA on EGFR clustering in *Drosophila* using STED microscopy. For this purpose, a chimeric EGFR protein containing human extracellular and *Drosophila* intracellular domains (62) was expressed in ISCs. This model allows for the labeling of EGFR in intact gut ISCs in a manner compatible with STED imaging (supplemental Fig. S8A–E). Utilizing the same dietary enrichment strategy as previously described, flies fed a DHA-enriched diet as compared with OA and low PUFA controls exhibited reduced EGFR cluster size (Fig. 4E–I). Collectively, these results demonstrate the ability of DHA to attenuate EGFR cluster formation across multiple in vitro and in vivo models.

### PA, PIP_2_, and cholesterol maintain EGFR nanocluster formation

**Fig. 5:**
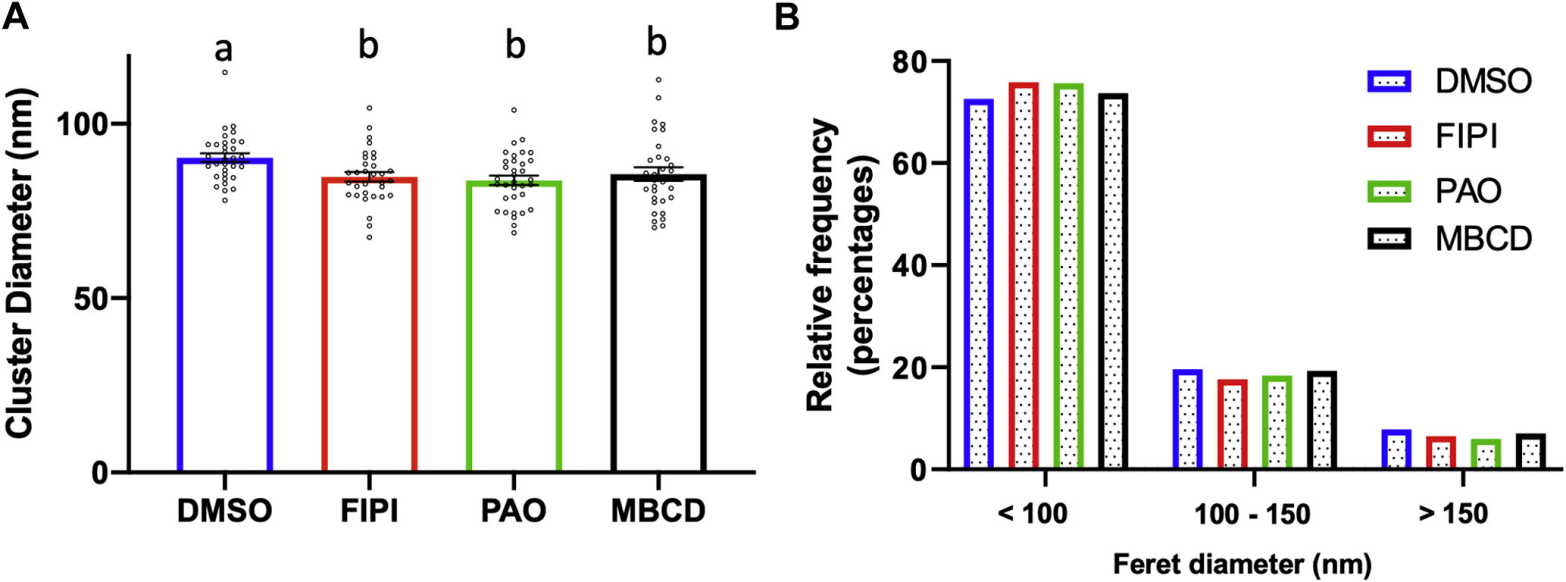
Influence of PIP_2_, PA, and cholesterol on EGFR nanocluster size in mouse colonic cells. YAMC cells were incubated with DMSO (0.1%), FIPI (1 μM), PAO (1 μM), or MβCD (10 mM) for 30 min at 33°C, before fixation and labeling with EGF-Alexa647 for STORM imaging. A: Quantitative analysis of EGFR cluster diameter and (B) relative frequency in YAMC cells. Data are presented as (A) mean ± SE of average EGFR cluster diameter per FOV and (B) individual cluster size distribution. Number of FOVs examined per treatment, DMSO = 32, FIPI = 32, PAO= 34, and MβCD = 31, and individual clusters, DMSO = 3,559, FIPI = 2,443, PAO = 1,512, and MβCD = 2,700, from 3 wells per group from 3 independent experiments. Statistical significance between treatments as indicated by uncommon letters (*P* < 0.05) was analyzed using one-way ANOVA and uncorrected Fisher's LSD tests.

The acidic lipids PA and PIP_2_ as well as cholesterol are key structural components of EGFR signaling proteolipid nanodomains (22, 23, 24, 63, 64, 65, 66, 67). To investigate the role of PA, PIP_2_, and cholesterol in EGFR nanocluster formation in our models, we treated YAMC cells with 5-fluoro-2-indolyl des-chlorohalopemide (FIPI), phenylarsine oxide (PAO), or methyl-beta-cyclodextrin (MβCD) to reduce levels of PA, PIP_2_, and cholesterol, respectively (25, 36, 66, 67). As expected, the suppression of PA, PIP_2_, and cholesterol reduced the size of EGFR nanoclusters (Fig. 5A, B). In addition, we determined the functional consequences of inhibitor-mediated EGFR cluster size reduction. Downstream signaling, e.g., spatiotemporal Ras activation (supplemental Fig. S9A, B), was also inhibited by FIPI and PAO. Overall, these findings support the premise that EGFR nanocluster formation and signal propagation are in part dependent on lipid structural components.

### DHA disrupts EGFR proteolipid composition

**Fig. 6:**
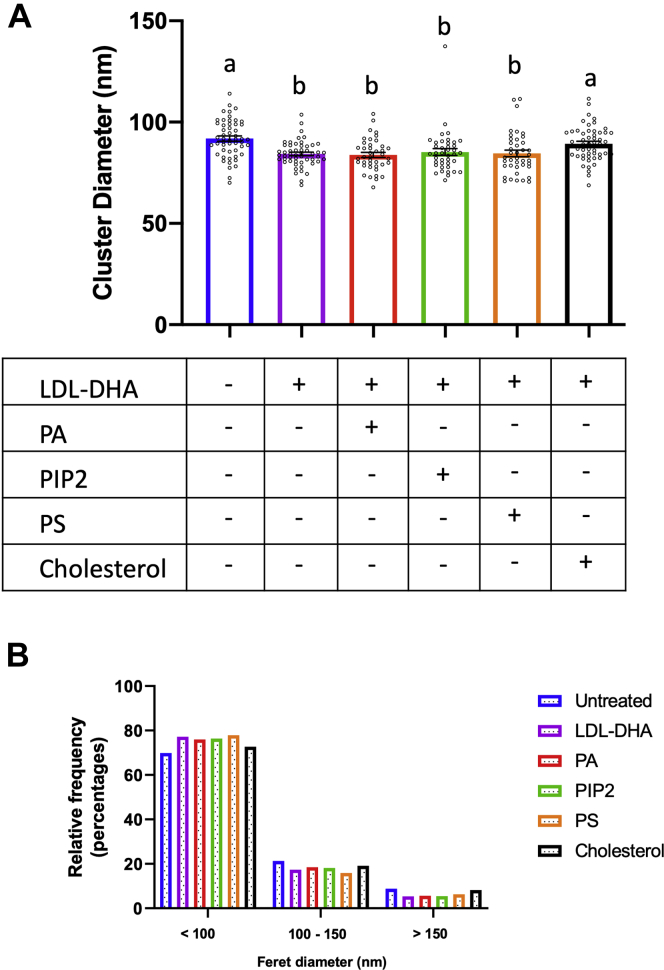
Exogenous cholesterol restores EGFR cluster formation in DHA-treated cells. YAMC cells were untreated or incubated with LDL-DHA (50 μM) for 24 h before the addition of media alone or media supplemented with PA (100 μM), PIP_2_ (100 μM), PS (100 μM), or cholesterol (1 mM) for 30 min at 33°C, before fixation and labeling with EGF-Alexa647 for STORM imaging. A: Quantitative analysis of EGFR cluster diameter and (B) relative frequency in YAMC cells. Data are presented as (A) mean ± SE of average EGFR cluster diameter per FOV and (B) individual cluster size distribution. Number of FOVs examined per treatment, untreated = 52, LDL-DHA = 52, LDL-DHA + PA = 40, LDL-DHA + PIP_2_ = 40, LDL-DHA + PS = 40, LDL-DHA + cholesterol = 52, and individual clusters, untreated = 6,196, LDL-DHA = 5,435, LDL-DHA + PA = 3,451, LDL-DHA + PIP_2_ = 2,319, LDL-DHA + PS = 2,870, LDL-DHA + cholesterol = 5,790, from five wells per group from three independent experiments. Statistical significance between treatments as indicated by uncommon letters (*P* < 0.005) was analyzed using one-way ANOVA and uncorrected Fisher's LSD tests.

Because PA, PIP_2_, and cholesterol play an important role in maintaining EGFR clustering and signaling, we hypothesized that exogenous addition of these components would rescue EGFR clustering in the presence of DHA. Of note, only the addition of cholesterol, but not PA or PIP_2_, restored EGFR cluster formation (Fig. 6A, B; supplemental Fig. S10). We have previously established that DHA can displace EGFR from cholesterol-rich lipid raft domains (15). In the present study, we use fluorescence lifetime imaging combined with fluorescence resonance energy transfer (FLIM-FRET) microscopy to monitor nanoscale (<10 nm) interactions between fluorescently tagged EGFR and a cholesterol binding probe (68, 69). When coexpressed with a corresponding FRET pair, such as red fluorescent protein, a reduction of GFP lifetime is indicative of more extensive FRET as a result of a smaller distance between GFP and red fluorescent protein, which is correlated with more extensive nanoclustering. Lifetime values were then converted to apparent FRET efficiency %, where an increase is indicative of enhanced nanoclustering (70). Of note, YAMC cells treated with DHA showed a reduction in FRET efficiency between EGFR and cholesterol (supplemental Fig. S11). These observations indicate that DHA-mediated reduction of EGFR clustering involves cholesterol-EGFR interactions.

## Discussion

There is a critical need for the development of novel anti-EGFR targeted therapies as patients utilizing current therapies often exhibit undesirable side effects (5) or develop acquired resistance (6, 7). A large body of epidemiological, preclinical, and clinical evidence supports the role of DHA as an adjuvant therapy for colon cancer (12, 13, 14, 15, 16, 17, 18). However, if DHA is to be pursued as an alternative/complementary strategy for anti-EGFR therapy, the mechanism by which it suppresses EGFR signaling must be elucidated.

In this study, we utilized complementary super-resolution microscopy techniques to demonstrate that cellular membrane phospholipid enrichment of DHA by therapeutic nanoparticle delivery, endogenous synthesis, or diet suppresses EGFR-mediated phenotypes and reduces EGFR nanocluster formation across a variety of in vitro and in vivo models. This is noteworthy, because EGFR nanocluster formation influences the efficiency of signal propagation (22, 23, 25). Dimerization and subsequent phosphorylation of EGFR does not sufficiently induce ERK phosphorylation (21, 71). These observations are consistent with reports that DHA-induced hyperphosphorylation of EGFR paradoxically suppressed Ras activation and ERK phosphorylation by reducing Ras/Sos1 interaction (15, 16). The mechanism by which EGFR nanocluster formation supports signal propagation is still not fully understood; however, it likely involves interactions with Ras, Sos1, and critical structural lipids (25, 72). Sos1 is recruited to the plasma membrane by activated EGFR, which subsequently engages Ras. This process is subject to complex regulation by the temporal and spatial production of phosphoinositides through interactions with SOS1 Dbl homology/pleckstrin homology regulatory domains (73). The acidic lipids PA and PIP_2_ regulate Sos1 recruitment to the plasma membrane where it interacts with and activates Ras to propagate downstream signaling (64, 74, 75, 76). Furthermore, PA production by phospholipase D2 and the nanoscale organization of Ras are influenced by cholesterol (77, 78, 79, 80). Overall, this supports a model where the formation of EGFR nanoclusters is driven by lipid-protein interactions whose proteolipid composition acts as a niche to recruit the appropriate components necessary to activate Ras and propagate downstream signaling. Our data indicate that the removal of any one of these lipid components is sufficient to reduce EGFR cluster formation, which is consistent with previous reports (22, 23).

Here, we extend previous studies focusing on membrane biophysical properties by addressing the impact of DHA on the nanoscale structure of EGFR. With regard to the molecular mechanism by which DHA reduces EGFR cluster size, we focused on lipids that serve as key structural components of EGFR signaling proteolipid nanodomains. We previously reported that DHA influences both PA and PIP_2_-related protein interactions (17, 81). However, neither the exogenous addition of PA or PIP_2_ restored DHA-induced EGFR cluster reduction. Instead, only cholesterol supplementation restored EGF-dependent EGFR clustering. This is supported by the FLIM-FRET-based observation that DHA reduces the interaction of EGFR and cholesterol occurring at the <10 nm scale (supplemental Fig. S11). Furthermore, the mutual aversion between DHA-containing phospholipids and cholesterol is well documented (48, 82, 83). In regard to how exogenous cholesterol restores EGFR cluster formation, we posit that the high levels of exogenous cholesterol simply overcome the biophysical effects of DHA. Another possibility is that cholesterol restores the interaction of PA and PIP_2_ with EGFR. Further work is needed to address this putative model.

EGFR nanoclusters are reported to contain ∼5–30 receptors and range in size from ∼20 to 630 nm depending on the methodology used (21, 22, 23, 24, 84, 85, 86). Similar to the reported size for EGFR clusters, we observed that individual EGFR clusters occur on a large spectrum ranging from ∼40 to 700 nm. Of note, the effect of DHA on average EGFR cluster size was modest (∼10%) across all models; however, the effect of DHA on large clusters (>150 nm) was more pronounced (Fig. 4). We speculate that these larger clusters may be the most efficient activators of Ras. This is noteworthy, because small (<10 nm) changes to EGFR organization driven by EGFR-lipid interactions may influence effector recruitment similar to what occurs with Ras signaling (22, 23, 68, 79, 80, 87).

The ability of DHA to reduce membrane rigidity is conserved across many cell types (33, 88, 89). This is likely driven by the incompatibility of DHA and major lipid raft components such as cholesterol and sphingomyelin (45, 46, 48, 82), as both these components play a large role in influencing plasma membrane biophysical properties as well as EGFR activity (90). Altering the biophysical properties of the plasma membrane may have broad effects on many membrane proteins. Although this study focused only on EGFR, DHA is known to affect the nanoscale architecture of other membrane proteins including Ras, Akt, Lck, and Lat (17, 54, 81, 91). Therefore, it is possible that the functionality of other membrane receptors may be impacted. Although assessment of membrane rigidity provides information on the structure of the membrane itself, at present, it is difficult to predict its association with protein signaling. This is because the biophysical properties of the plasma membrane such as rigidity are the result of a myriad of variables, including lipid saturation, cholesterol content, lipid-protein interactions, and cytoskeletal dynamics (36, 57, 58). Similarly, we cannot rule out the contribution of a DHA bioactive metabolite, although to date there is no evidence that secreted lipid metabolites (in general) have membrane-altering properties. Therefore, additional work is needed to determine if DHA impacts the nanoscale architecture of other EGFR mutants that arise from anti-EGFR therapies (92), including the clinically relevant EGFR family member HER2 (93, 94).

Although experimental, epidemiological, preclinical, and clinical evidence support a protective benefit of DHA against colon and breast tumorigenesis (12, 95, 96), this may not be true for all cancers. Recently, it was reported that ELOVL2, an elongase that functions in the synthesis of the long-chain n-3 and n-6 PUFAs, e.g., DHA and docosapentaenoic acid (DPA, 22∶5^Δ4,7,10,13,16^), respectively, is required for the maintenance of glioblastoma stem cells and that dual targeting of PUFA synthesis and EGFR signaling had a combinatorial cytotoxic effect of glioblastoma stem cells (97). The mechanism underlying the unique effects of DHA in these two tissues is not understood but may involve how EGFR nanocluster proteolipid composition is maintained in each instance. Although in most cases, DHA is relatively nontoxic to nontransformed cells, the previous example highlights the benefit of selective targeting of DHA. This is consistent with previous reports that DHA specifically targets tumor subtypes (26, 98), attenuating EGFR nanoclustering.

In conclusion, our novel findings suggest that the ability of DHA to reduce EGFR nanocluster formation plays a crucial role in the attenuation of hyperactive EGFR-driven signaling and phenotypes. These findings support the feasibility of using dietary strategies that target plasma membrane nanoscale architecture in order to reduce oncogenic signaling and cancer risk.

### Data availability

All data reported in this study are located within the article.

## Conflict of interest

The authors declare that they have no conflicts of interest with the contents of this article.
