# Supplementary material for: Membrane therapy using DHA suppresses epidermal growth factor receptor signaling by disrupting nanocluster formation
Source: J Lipid Res. 2021 Jan 27;62:100026. doi: 10.1016/j.jlr.2021.100026 (PMC7933808; doi:10.1016/j.jlr.2021.100026)
Supplement: Supplemental Information [file mmc1.pdf]

Supplemental Information

Title: Membrane therapy using DHA suppresses epidermal growth factor receptor signaling by disrupting nanocluster formation

Authors and affiliations: Natividad R. Fuentes[1,2,3], Mohamed Mlih[4], Xiaoli Wang[1,2], Gabriella Webster[1,2], Sergio Cortes-Acosta[1,2], Michael L. Salinas[1,2], Ian R. Corbin[5], Jason Karpac[4] and Robert S. Chapkin[1,2,3,6].

[1]Program in Integrative Nutrition & Complex Diseases; [2]Department of Nutrition, [3]Interdisciplinary Faculty of Toxicology; [4]Department of Molecular and Cellular Medicine, College of Medicine, Texas A&M Health Science Center; [5]Advanced Imaging Research Center, University of Texas Southwestern Medical Center at Dallas and [6]Center for Translational Environmental Health Research, Texas A&M University.

Methods

Drosophila Low PUFA Diet

A low PUFA diet was prepared as previously described (1). Briefly, individual diet components were mixed and prepared in a gel form with agar. Once the diet cooled to < 60°C, OA (0.5% w/w) or DHA (0.5% w/w) was added and the diet was vigorously shaken to ensure uniform mixing, prior to decanting into plastic feeding tubes. Newly emerged adult female flies were transferred to diets for 5 d at 18°C then 2 d at 29°C prior to dissection and the collection of their guts for imaging. Dietary DHA was well tolerated, which is consistent with previous studies demonstrating that dietary long-chain fatty acids are tolerated and readily incorporated into larvae, pupae and adult fly membrane lipids (2–4).

Conditional Expression of UAS-Linked Transgenes

The esgGal4 driver was combined with a ubiquitously expressed temperature-sensitive Gal80 inhibitor (tubGal80ts). The escargot construct drives expression in stem cells and enteroblast. Crosses and flies were kept at 18°C (permissive temperature) and 5 d-old females were fed the test diets 5 d and then shifted to 29°C for 2 d to allow expression of the transgenes before analysis.

Drosophila Immunostaining and Microscopy

Intact fly guts were fixed at room temperature for 20 min in 100 mM glutamic acid, 25 mM KCl, 20 mM $MgSO_4$, 4 mM sodium phosphate, 1 mM $MgCl_2$, and 4% formaldehyde. All subsequent incubations were performed using PBS, 0.5% BSA, 0.1% Triton X-100 at 4°C. To assess proliferation guts were incubated with primary anti-phospho-Histone H3 (Cell Signaling #9701S; 1:1000) was applied overnight at 4 °C. Alexa Fluor-conjugated secondary (Jackson Immunoresearch, 1:500) antibodies were incubated for 2 h at room temperature. Hoechst was used to stain DNA. Guts were mounted in Mowiol medium and visually scanned using a Leica M165FC fluorescent stereo microscope to count pH3 positive cells per gut. Representative images were collected using a Leica TCS SPE confocal system. To assess phospho-ERK levels in esgG4 cells guts were incubated with primary phospho-ERK (Cell Signaling Technology, #4370, 1:200, RRID:AB_2315112) antibody followed by CF640R fluorescent secondary antibody (Biotium Cat# 20178, 1:400, RRID:AB_10854111).Hoechst dye was used to stain DNA. Guts were mounted in Mowiol medium and imaged with a Leica confocal microscope using a 40x 1.15 NA oil objective. A Z-stacks of approximately 5-10 planes at 5 µm steps spanning the gut was acquired. For analysis, images were opened in NIH ImageJ software (ImageJ, RRID:SCR_003070; Fiji, RRID:SCR_002285), converted to Tiff files and a custom macro was used to quantify average fluorescent intensity of pERK in esgG4 cells. Briefly, a maximum image projection was generated, and esgG4-GFP was used to define a binary cell mask that was applied to pERK images. Average fluorescent intensity of the mask images was subsequently recorded (**supplemental Fig. S12**).

d-STORM Super Resolution Microscopy Imaging and Nanocluster Analysis

Samples were prepared for STORM utilizing a Cysteamine (MEA) glucose oxidase (Glox) buffer described previously (5). Briefly, TN buffer (50 mM Tris (pH 8.0) and 10 mM NaCl), an oxygen scavenging system (0.5 mg ml^{-1} glucose oxidase (Sigma, G2133), 40 µg ml^{-1} catalase (Sigma, C40) and 10% (w/v) glucose) and 10 mM MEA (Sigma, 30070). MEA was stored as a solid at 4 °C and prepared fresh as a 1 M stock solution in 0.25 N HCL. This stock solution was kept at 4 °C and used within 2 wks of preparation.

STORM imaging and analysis was performed using a Nikon N-STORM 4.0 Super-Resolution System. Samples were imaged at room temperature in 8 chamber coverglass slides (Cellvis, C8-1.5H-N) by direct STORM on an inverted N-STORM 4.0 Super-Resolution System (Nikon Ti, Japan) equipped with an Apochromat TIRF 100×/1.49 NA oil immersion objective and a quad-band pass dichroic filter (Nikon, 97335). Images were then acquired in highly inclined and laminated optical sheet (HILO) microscopy mode and focal drift was prevented with hardware autofocusing (Nikon Perfect Focus System 4). Alexa Fluor 647 was pushed into the dark state using the 640 nm (125 mW) laser line at maximum laser intensity. Drift correction was performed using Nikon software. Data were acquired with a field-of-view FOV 256 × 256 pixels (160 nm pixel size), at 8.5 ms frame rate for 10,000 frames with an EMCCD camera (Princeton Instruments, ProEM-HS 512BX3). The sparsity of single molecules per frame was controlled with 405 nm laser (100 mW) at 20-60% of total power. Single molecule localization analysis was performed using Nikon N-STORM software. The Maximum Width, which is the maximum possible width a spot of some intensity in the image to be identified as a molecule by STORM Analysis was set to 400 nm. To exclude the identification of two molecules in close proximity, fluorescent spots whose ratio of elongation in the X and Y direction is larger than 1.3 were rejected as a single molecule. Lateral drift was compensated for using autocorrelation-based drift correction with NIS Elements software which corrects drift based on tracking the entire set of molecules.

For nanocluster measurements, nanoclusters were identified by Voronoi analysis in the NIS Elements software which is based on 3D Delaunay triangulation (6). Briefly, Voronoi creates clusters from the molecules present in the current image with the specified maximum distance between the adjacent molecules and the specified minimum number of molecules in the emerging cluster. The maximum distance was set at 50 nm, and minimum number of molecules at 10. A masked image of the clusters was exported to ImageJ, where it was converted to binary, and the analyze particle function was used to quantify clusters larger than 0.001 μm^2 (**supplemental Fig. S3**).

STED Super Resolution Microscopy Imaging and Nanocluster Analysis

Gated STED (gSTED) improves resolution and contrast over continuous wave (cw) STED (7). Gated STED imaging was conducted on a Leica SP8 g-STED 775 equipped with a white light (WLL) pulsed laser source and HyD hybrid detectors. Guts mounted in Mowiol medium were imaged at room temperature with an HC PL APO 100x/1.40 oil

immersion objective. Image planes were selected containing esgG4 cells that were closest to the glass coverslip. To obtain maximum optical resolution the 775 nm STED depletion beam was set at 100%. Time-gated detection with a gate of 1.5-6.0 ns was applied. Line averaging was set at 4 and frame accumulation at 8. Image acquisition was set at 1128 x 1128 pixels with 10 nm pixel size and 8-bit pixel depth. Scanning was unidirectional at 1000 Hz. Excitation was set at 590 nm with WLL, and emission collected with HyD detectors between 605-700 nm. For nanocluster measurements, STED images were converted into binary maps by applying a median filter (r = 1), difference of Gaussians filter (sigma 1 = 6, sigma 2 = 3) and an automatic triangle threshold using ImageJ. To avoid false detections caused by noise fluctuations or unbound labeled antibodies, objects with an area smaller than 0.001 μm^2 were excluded from further analysis.

Lipid Extraction and Fatty Acid Analysis

For assessment of lipid incorporation into membrane phospholipids, total lipids were extracted from YAMC cells with 2:1 (v/v) chloroform-methanol as previously described (8). Total phospholipids were subsequently separated by thin-layer chromatography with 90:8:1:0.8 (v/v/v/v) chloroform-methanol-acetic acid-water. After transesterification using methanolic HCL, fatty acid methyl esters were quantified by capillary gas chromatography-mass spectrometry (8).

Lipid Add-Back

18:0-18:1 phosphatidic acid (#840861, 10 mg/mL in chloroform), brain PIP_2 (#840046, 1 mg/mL in chloroform) and 18:0-18:1 phosphatidylserine (#840039, 10 mg/mL in chloroform) were obtained from Avanti Polar Lipids. Water soluble cholesterol (#C4951) was obtained from Sigma. PA, PIP_2 and PS were dried under nitrogen gas, and resuspended to 10 mM in 150 mM NaCl and 10 mM Tris·HCl, pH 8, and immediately added to cultures at 100 µM final concentration in RPMI. Cholesterol was resuspended to 10 mM in RPMI, and immediately added to cultures at 1 mM final concentration in RPMI. All lipids were incubated for 30 min at 33°C prior to cell fixation. Where indicated, SW48 cells were transfected with plasmid encoding the PIP_2 binding sensor (PLC-PH-GFP) or PA binding sensor (Spo20-GFP) 24 h prior to incubation with lipids as described above. The change in the plasma membrane localization was quantified using the line pixel intensity histogram in NIH Image J as described previously (9). Briefly, a line was randomly drawn to span a large

area of the cytoplasm but not the nucleus. The pixel intensity of the two most outside peaks represented the biosensor on the plasma membrane, while the average value between represented that in the cytoplasm.

Fluorescence Lifetime Imaging Microscopy combined with Fluorescence Resonance Energy Transfer (FLIM-FRET)

YAMC cells were seeded at a density of 7.5×10^3 in cell imaging 8 chamber coverglass slides (Eppendorf, 0030742036) for 36 h prior to transfection using Lipofectamine 3000 (ThermoFisher, #L3000-008). Cells were transfected with plasmids of donor (EGFR-GFP) alone and/or with FRET acceptor (D4H-mCherry). FRET acceptors were mCherry-tagged proteins of interest (GFP: mCherry-plasmids at 1:2 ratio, 0.3 µg total plasmid per well). After 6 h in transfection media, cells were gently washed with PBS and incubated with complete media for an additional 24 h. For the final 24 h, complete media plus fatty acids (50 µM) were added. Subsequently, cells expressing GFP-tagged protein alone or co-expressing both GFP-tagged and mCherry-tagged proteins were washed with PBS and fixed in 4% PFA for 15 min. After three washes in 1X PBS, HPBS was added to wells. The GFP fluorescence lifetime was measured using a Lambert Instrument (Roden, The Netherlands) FLIM unit attached to a wide field Nikon Eclipse microscope. GFP was sinusoidally excited by a modulating 3-Watt 497 nm light-emitting diode (LED) at 40 MHz under epi-illumination. A solution of fluorescein (1 µM) was used as a lifetime reference standard. Cells were imaged with a 1.3 numerical aperture 40x Plan-Fluor oil objective using an appropriate GFP filter set. The phase and modulation were determined from 12 phase settings using the manufacturer's software. This analysis resulted in an image where the fluorescence lifetime of GFP was determined and assigned to each pixel. The color scale on each pixel represents the fluorescent lifetime, which equates to the level of interaction between the GFP and RFP tagged proteins. Lifetime (phase) values were pooled and averaged from regions of interest drawn on individual cells. Each experiment was replicated at least 3 times. The FLIM-FRET method is highly favored over intensity based FRET measurements because fluorescent lifetime is an intrinsic property of the fluorescent molecule and is generally insensitive to weak signal, excitation source, and variations in the donor-acceptor ratio (10). The percentage of the apparent FRET efficiency (E_{app}) was calculated using the measured lifetimes of each donor-acceptor pair (τ_{DA}) and the average lifetime of the donor only (τ_D) samples. The formula employed was $Eapp = (1-\tau_{DA}/ \tau_D) \times 100\%$ (11).

Supplemental References:

1. Ziegler AB, Ménagé C, Grégoire S, Garcia T, Ferveur J-F, Bretillon L, et al. Lack of Dietary Polyunsaturated Fatty Acids Causes Synapse Dysfunction in the Drosophila Visual System. Mollereau B, editor. PLoS One [Internet]. 2015 Aug 26 [cited 2019 Oct 3];10(8):e0135353. Available from: http://www.ncbi.nlm.nih.gov/pubmed/26308084

2. Carvalho M, Sampaio JL, Palm W, Brankatschk M, Eaton S, Shevchenko A. Effects of diet and development on the Drosophila lipidome. Mol Syst Biol [Internet]. 2012 Jul 31 [cited 2015 Oct 4];8(600):600. Available from: http://www.pubmedcentral.nih.gov/articlerender.fcgi?artid=3421444&tool=pmcentrez&rendertype=abstract

3. Fuentes NR, Mlih M, Barhoumi R, Fan Y-Y, Hardin P, Steele TJ, et al. Long-Chain n-3 Fatty Acids Attenuate Oncogenic KRas-Driven Proliferation by Altering Plasma Membrane Nanoscale Proteolipid Composition. Cancer Res [Internet]. 2018 Jul 15 [cited 2018 Oct 31];78(14):3899–912. Available from: http://www.ncbi.nlm.nih.gov/pubmed/29769200

4. Shen LR, Lai CQ, Feng X, Parnell LD, Wan JB, Wang JD, et al. Drosophila lacks C20 and C22 PUFAs. J Lipid Res [Internet]. 2010 Oct [cited 2015 Sep 14];51(10):2985–92. Available from: http://www.pubmedcentral.nih.gov/articlerender.fcgi?artid=2936753&tool=pmcentrez&rendertype=abstract

5. Dempsey GT, Vaughan JC, Chen KH, Bates M, Zhuang X. Evaluation of fluorophores for optimal performance in localization-based super-resolution imaging. Nat Methods [Internet]. 2011 Dec 6 [cited 2019 Dec 5];8(12):1027–36. Available from: http://www.ncbi.nlm.nih.gov/pubmed/22056676

6. Levet F, Hosy E, Kechkar A, Butler C, Beghin A, Choquet D, et al. SR-Tesseler: a method to segment and quantify localization-based super-resolution microscopy data. Nat Methods [Internet]. 2015 Nov 7 [cited 2019 Dec 9];12(11):1065–71. Available from: http://www.ncbi.nlm.nih.gov/pubmed/26344046

7. Vicidomini G, Moneron G, Han KY, Westphal V, Ta H, Reuss M, et al. Sharper low-power STED nanoscopy by time gating. Nat Methods [Internet]. 2011 Jul 5 [cited 2019 Dec 6];8(7):571–3. Available from: http://www.ncbi.nlm.nih.gov/pubmed/21642963

8. Fan YY, McMurray DN, Ly LH, Chapkin RS. Dietary (n-3) polyunsaturated fatty acids remodel mouse T-cell lipid rafts. J Nutr [Internet]. 2003;133(6):1913–20. Available from: http://www.ncbi.nlm.nih.gov/pubmed/12771339

9. Balla T, Várnai P. Visualization of cellular phosphoinositide pools with GFP-fused protein-domains [Internet]. Vol. Chapter 24, Current Protocols in Cell Biology. Curr Protoc Cell Biol; 2009 [cited 2020 Aug 4]. Available from: https://pubmed.ncbi.nlm.nih.gov/19283730/

10. Chang C, Sud D, Mycek M. Fluorescence Lifetime Imaging Microscopy. In: Methods in cell biology [Internet]. 2007 [cited 2018 Mar 19]. p. 495–524. Available from: http://www.ncbi.nlm.nih.gov/pubmed/17519182

11. Najumudeen AK, Jaiswal A, Lectez B, Oetken-Lindholm C, Guzmán C, Siljamäki E, et al. Cancer stem cell drugs target K-ras signaling in a stemness context. Oncogene [Internet]. 2016 [cited 2016 Jun 7];35(40):5248–62. Available from: http://www.nature.com/doifinder/10.1038/onc.2016.59

Supplemental figure and figure legends:

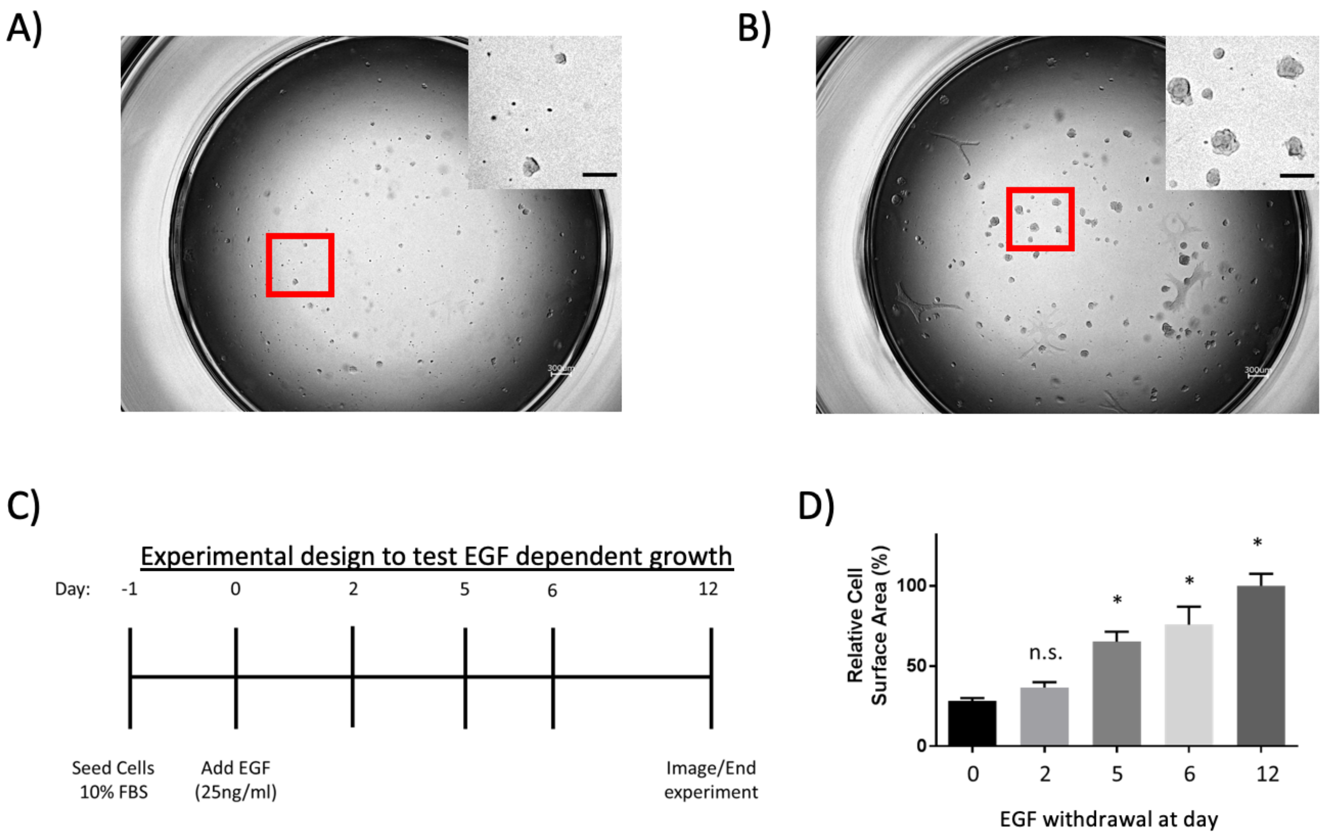

Supplemental Figure S1. Development of a 3D model of EGF-dependent growth. IMCE cells are immortalized murine colonic cells derived from crossing the Immortomouse with the MIN mouse carrying one mutant APC allele. The Immortomouse contains a temperature-sensitive mutation of the SV40 large T gene, an immortalizing gene that binds to p53 and inhibits its role in senescence. Representative images of 3D colonoids formed by IMCE cells grown in Matrigel™ (A) without and (B) with EGF (25 ng/mL) for 12 d. Inset scale bar, 200 µm. 1,500 IMCE cells in 50 µL Matrigel™ were seeded in a 96 well plate, with 150 uL complete media including IFN-γ and 10% FBS and ± EGF. Media was changed every 3-4 d. Representative images of wells grown after 12 d. IMCE cells formed budding organoid structures. Cells were imaged with a 2x objective on a Keyence microscope. Z-stacks of 30 planes at 20 µm steps were collected. Full focused images represent the combination of all stacks into one image. Scale bar, 300 µm. (C) Experimental design to assess EGF-dependent colonoid growth. 90 IMCE cells were seeded with 3 µL Matrigel in a 96-well plate, in RPMI supplemented with 10% FBS and IFN-γ at 33C°. EGF was withdrawn from the media at the indicated

day, and colonoids were allowed to grow until the end of the experiment at d 12, upon which (D) surface area was quantified.

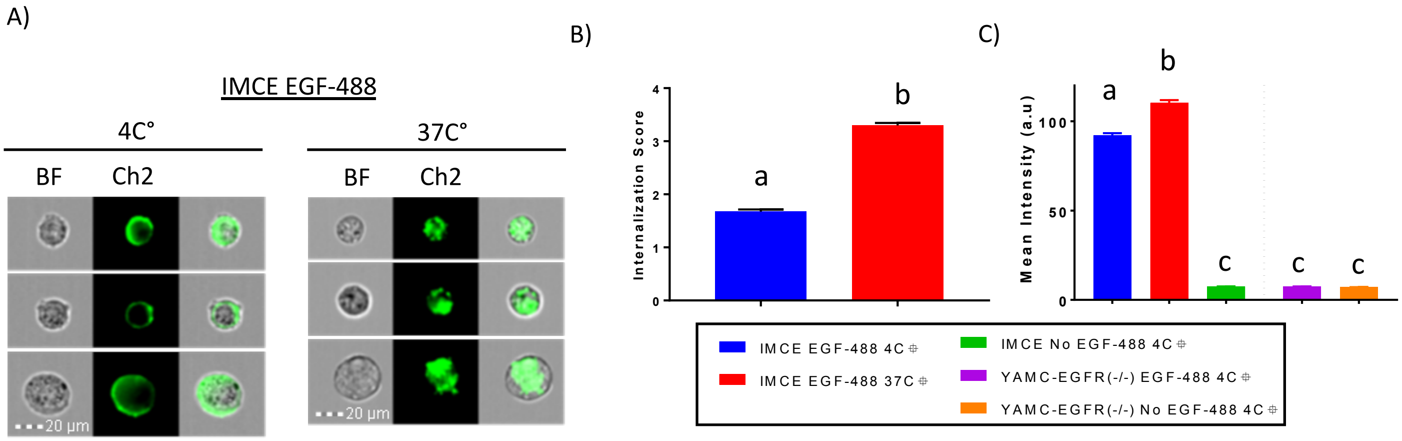

Supplemental Figure S2. Alexa Fluor conjugated EGF specifically labels plasma membrane bound EGFR. Resuspended IMCE and YAMC-EGFR (-/-) cells were incubated with 2 µg/mL EGF-Alexa488 for 30 min at the indicated temperature, followed by rinsing and live cell imaging performed using an Amnis Flowsight flow cytometer. (A) Representative bright-field (BF) and fluorescent (Ch2) images of IMCE cells incubated at 4°C and 37°C, demonstrating various degrees of EGFR internalization. (B) Quantification of EGFR internalization, where a high higher score reflects more internalization. (C) Quantification of mean fluorescent intensity from IMCE (EGFR expressing) and YAMC-EGFR (-/-) (Non-EGFR expressing) cells. As expected, fluorescence intensity of YAMC-EGFR (-/-) incubated with EGF-Alexa488 was similar to IMCE cells that were not incubated with EGF-Alexa488, indicating no presence of EGFR. Statistical significance between treatments as indicated by mean values with uncommon letters ($P < 0.0001$) was analyzed using a (B) unpaired t-test or (C) one-way ANOVA and Tukey multiple comparisons test. At least 925 cells were examined per group.

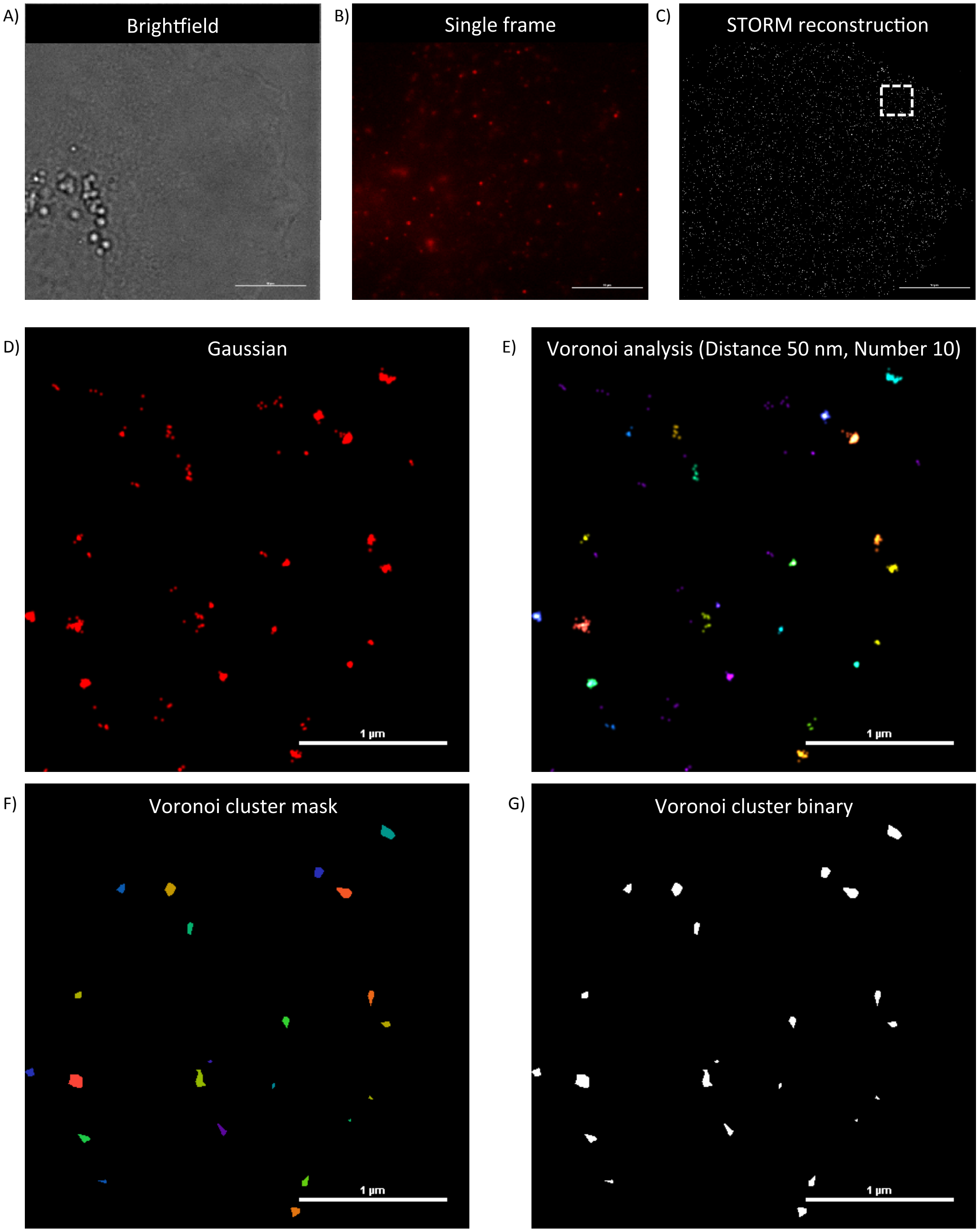

Supplemental Figure S3. STORM EGFR nanocluster image analysis pipeline. Representative (A) brightfield, (B) single frame fluorescence and (C) reconstructed STORM image generated from 10,000 frames. Scale bar, 10 μm. (D) Magnified view of reconstructed STORM image displayed as a Gaussian distribution centered on each molecule's coordinates. (E) A Voronoi analysis (diameter 50, number 10) was conducted on STORM localizations generating an

image where molecules assigned to a cluster share a color and molecules that are not assigned to a cluster also share a similar color. (F) A mask was then superimposed on identified clusters which was used to define a (G) binary cluster mask and to calculate cluster size. Scale bar, 1 μm.

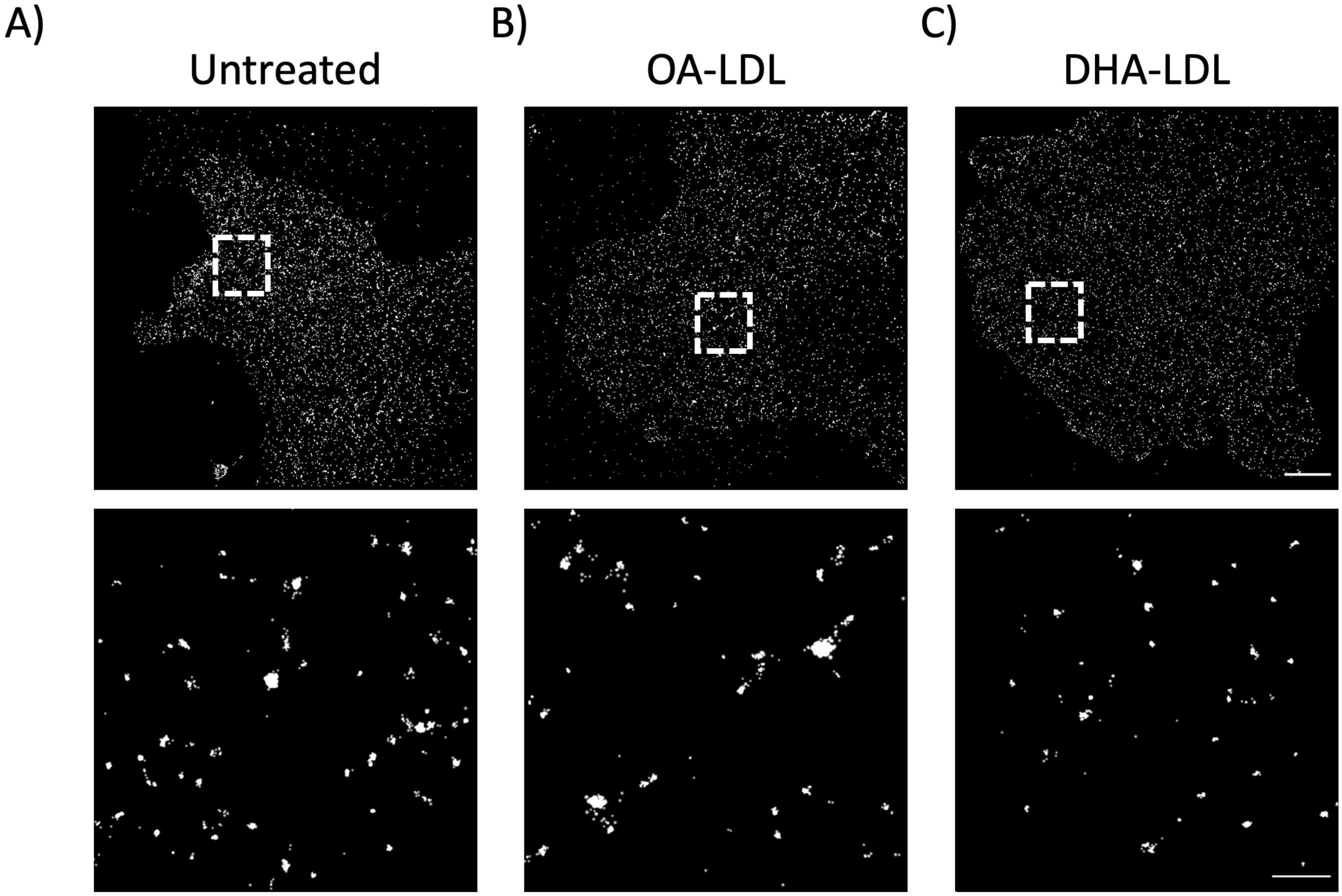

Supplemental Figure S4. Representative Storm images of YAMC cells. Representative STORM images of (A) untreated, (B) OA-LDL treated or (C) DHA-LDL treated cells quantified in Figures 4 A and B. Scale bar, 5 µm and 500 nm.

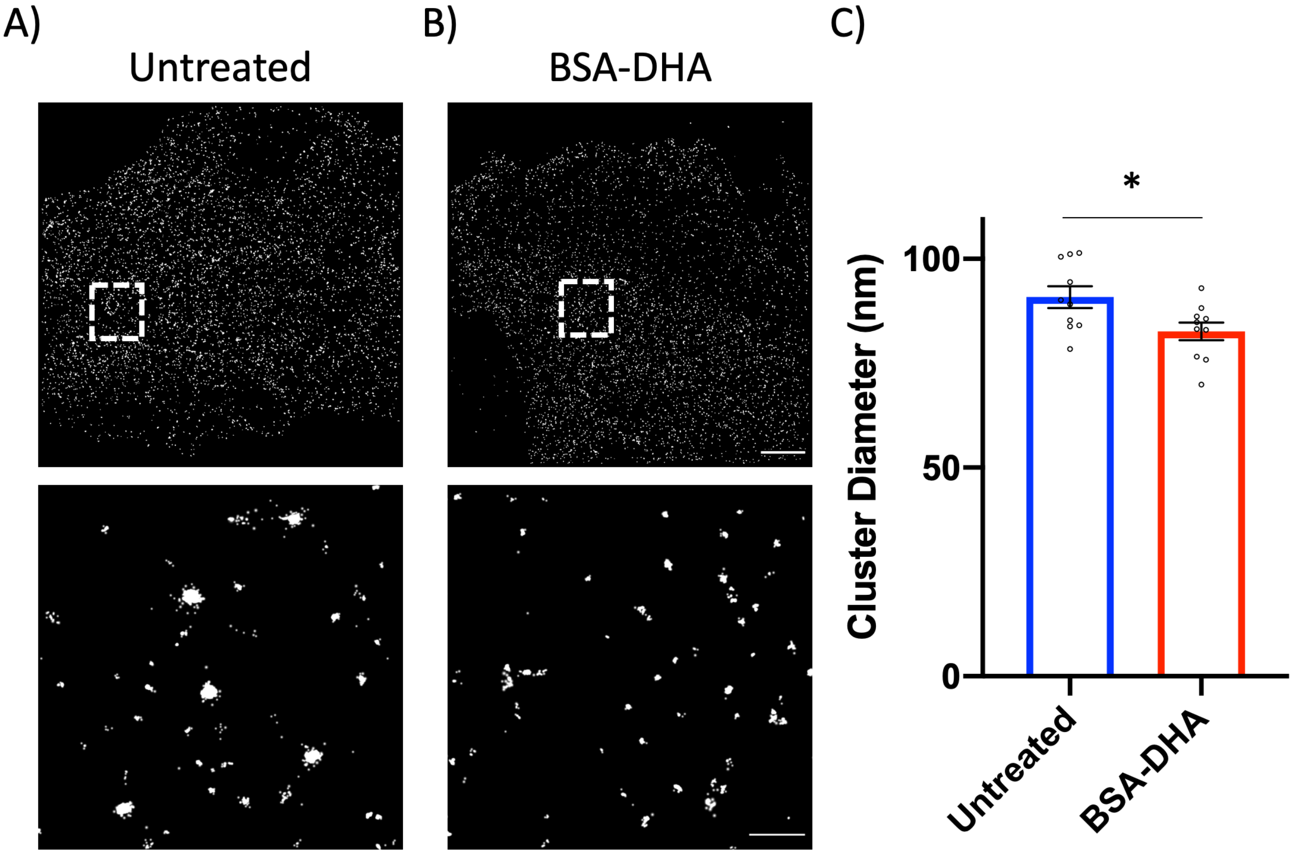

Supplemental Figure S5. BSA-DHA reduces EGFR nanocluster formation. YAMC cells were incubated with the indicated treatments (50 µM) for 24 h before fixation and labeling with EGF-Alexa647 for STORM imaging. Representative STORM images of (A) untreated and (B) BSA-DHA treated cells. Scale bar, 5 µm and 500 nm. (C) Quantitative analysis of EGFR cluster diameter. Data are presented as mean ± s.e. of average EGFR cluster diameter per field of view (FOV). Number of FOVs examined per treatment, untreated = 10 and BSA-DHA = 10. Statistical significance between groups (*$p < 0.05$) was analyzed using an unpaired t-test.

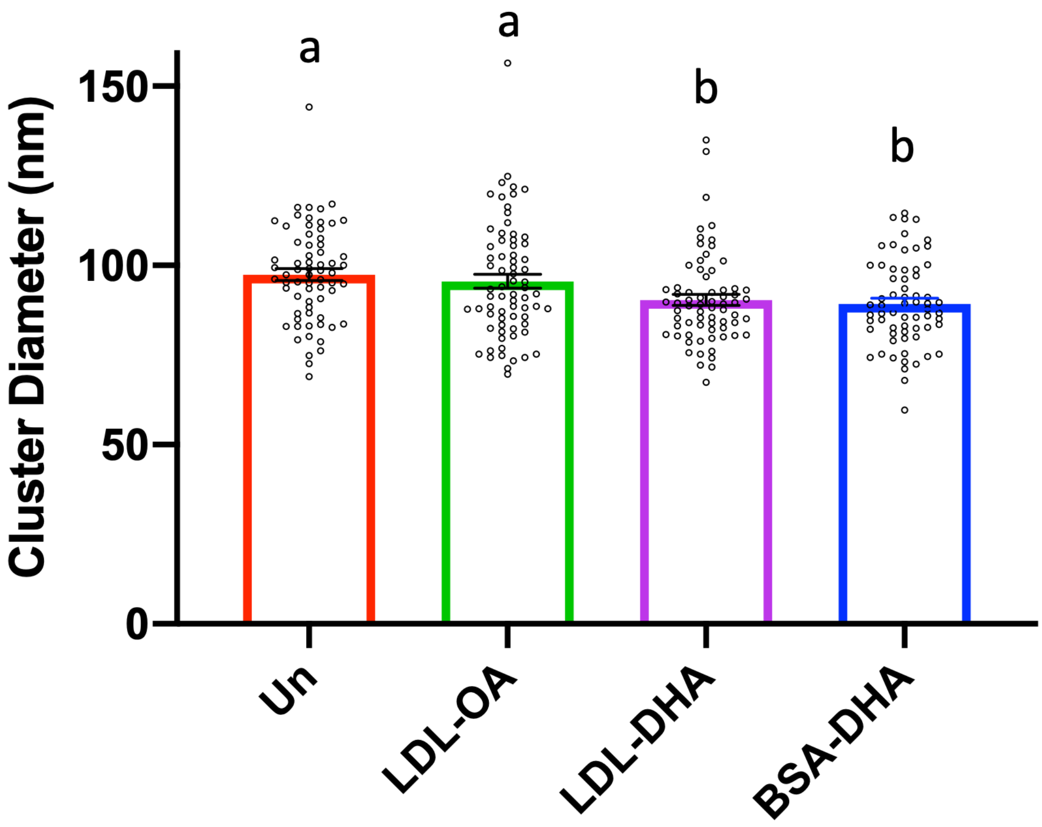

Supplemental Figure S6. DHA reduces mutant EGFR G719S nanocluster formation. SW48 cells were incubated with the indicated treatments (50 µM) for 24 h before fixation and labeling with EGF-Alexa647 for STORM imaging. Quantitative analysis of EGFR cluster diameter. Data are presented as mean ± s.e. of average EGFR cluster diameter per field of view (FOV). Number of FOVs examined per treatment, untreated = 67, OA-LDL = 71, DHA-LDL = 69 and DHA-BSA = 65. Statistical significance between groups as indicated by uncommon letters ($p < 0.01$) was analyzed using one-way ANOVA and uncorrected Fisher's LSD tests.

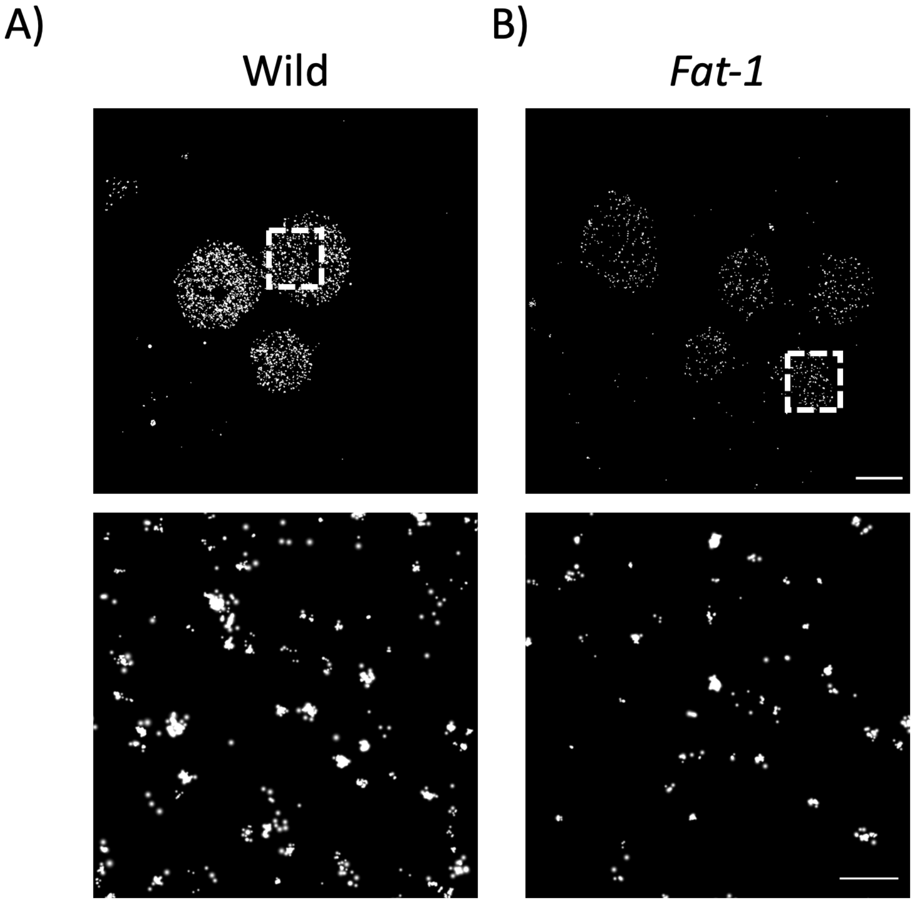

Supplemental Figure S7. Representative Storm images of isolated colonic cells. Representative STORM images of (A) wild type and (B) Fat-1 cells quantified in Figures 4 C and D. Scale bar, 5 µm and 500 nm.

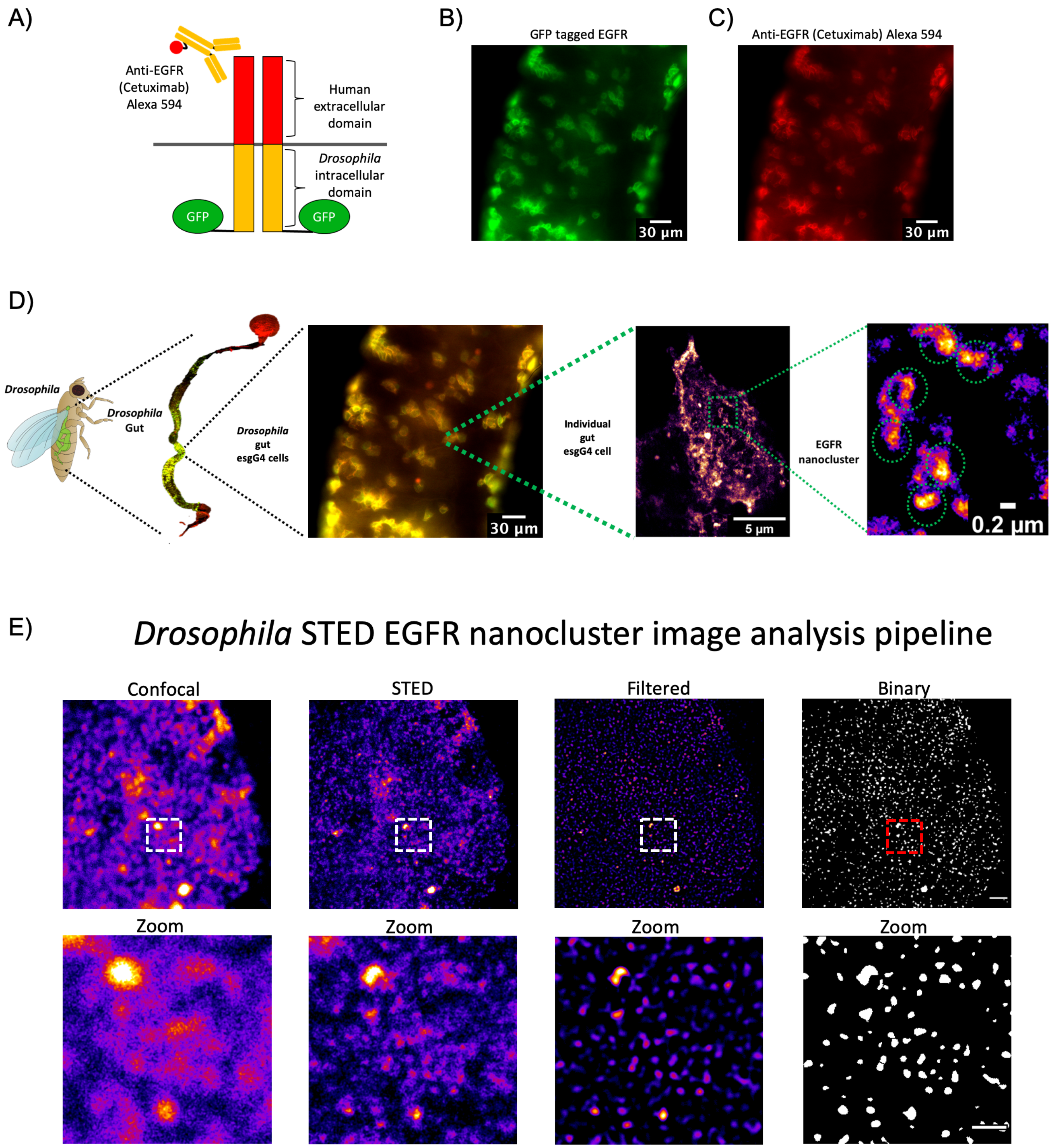

Supplemental Figure S8. *Drosophila* chimeric EGFR model for STED imaging. (A) Schematic diagram of chimeric EGFR (represented as a dimer) containing human extracellular and *Drosophila* intracellular domains tagged with GFP. Representative images of (B) EGFR-GFP and (C) cetuximab-Alexa 594 in *Drosophila* guts. (D) Depiction of a *Drosophila* gut showing clear overlap between EGFR-GFP with cetuximab-Alexa 594 and the presence of EGFR

nanoclusters in esgG4 cells. (E) STED EGFR nanocluster image analysis pipeline. Representative confocal, STED, filtered and binary images. Scale bar, 1 µm and 500 nm.

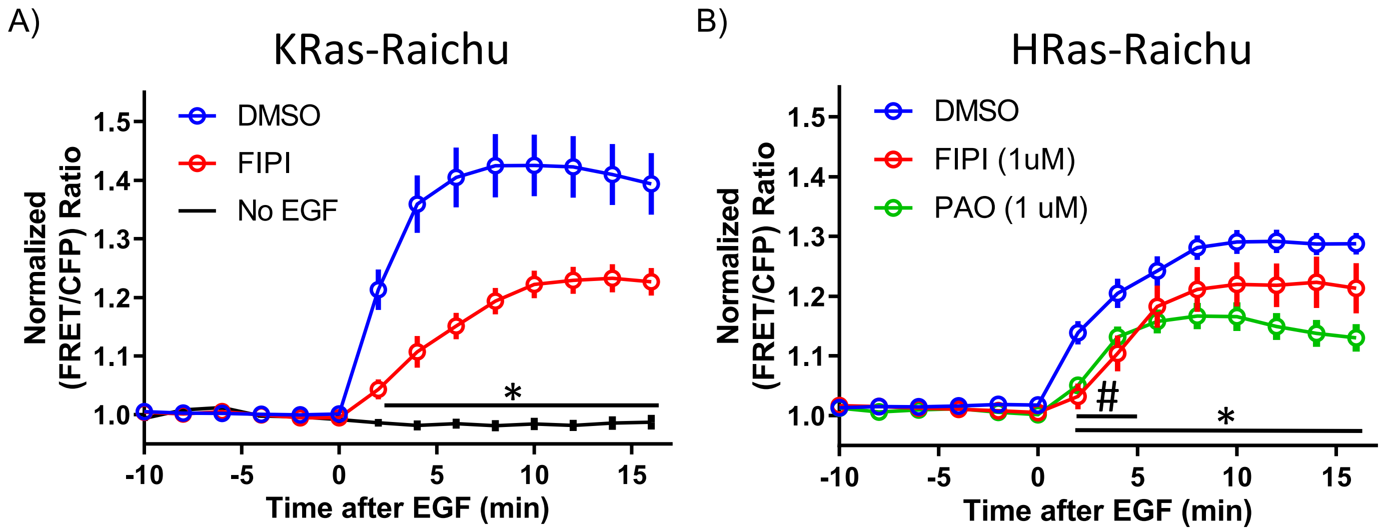

Supplemental Figure S9. Pharmacological reduction of PA and PIP_2 attenuates spatiotemporal Ras activation. Spatiotemporal activation of Ras was determined by monitoring activation of FRET biosensors targeted to (A) K- or (B) H-Ras domains. YAMC cells were transfected with plasmids encoding the indicated biosensor for 48 h, serum starved (0% FBS, 4 h) and incubated with DMSO (0.1%), FIPI (1 µM) or PAO (1 µM) for 30 min prior to stimulation with EGF (25 ng/mL) at 33C°. Images were taken at 60X magnification every 2 min. The net intensities of CFP and FRET in each cell were measured to calculate the averaged emission ratio (FRET/CFP). The FRET/CFP ratio was normalized by the average value before stimulation. (A) Data represent mean ± s.e., FRET ratio for each cell. Number of cells examined per treatment, DMSO = 7, FIPI = 14, and No EGF = 5. All points after 2 min are statistically significant ($p < 0.05$) between FIPI and DMSO (control) as indicated by bar and (*). (B) Data represent mean ± s.e., FRET ratio for each cell. Number of cells examined per treatment, DMSO = 33, FIPI = 13, and PAO = 35. The 2 and 4 min time points are statistically significant ($p < 0.05$) between FIPI and DMSO (control) as indicated by bar and (#). All points after 2 min are statistically significant ($p < 0.05$) between PAO and DMSO (control) as indicated by bar and (*).

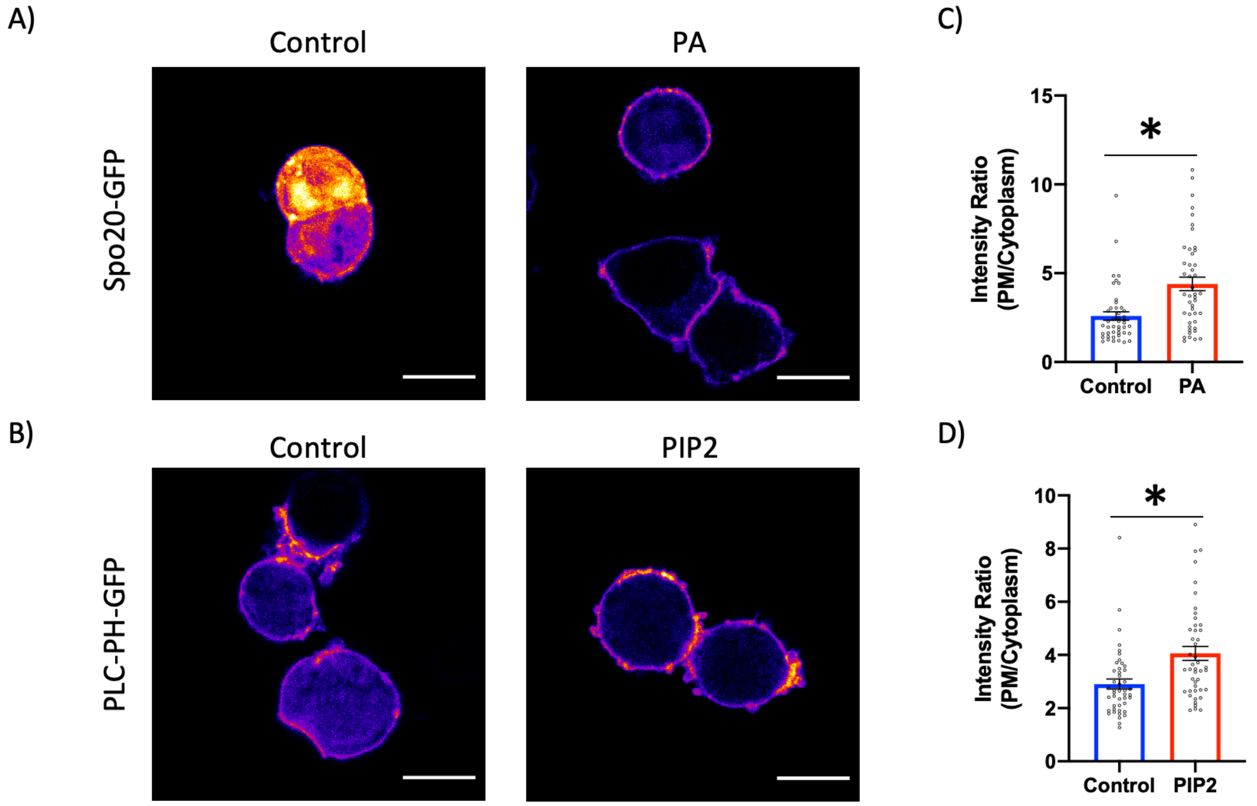

Supplemental Figure S10. PA and PIP_2 are enriched at the plasma membrane after exogenous supplementation. SW48 cells transiently expressing the indicated biosensor were incubated with 100 µM of indicated phospholipid for 30 min prior to fixation and confocal imaging. Representative confocal images of cells expressing (A) PA sensing Spo20-GFP or (B) PIP_2 sensing PLC-PH-GFP. Scale bar, 10 µm. (C and D) Quantitative analysis of plasma membrane enrichment of the indicated biosensor. Data are presented as mean ± s.e. of the average intensity ratio of PM/Cytoplasm per cell. A total of 45 cells were examined per treatment. Statistical significance between groups ($*p < 0.05$) was analyzed using an unpaired t-test.

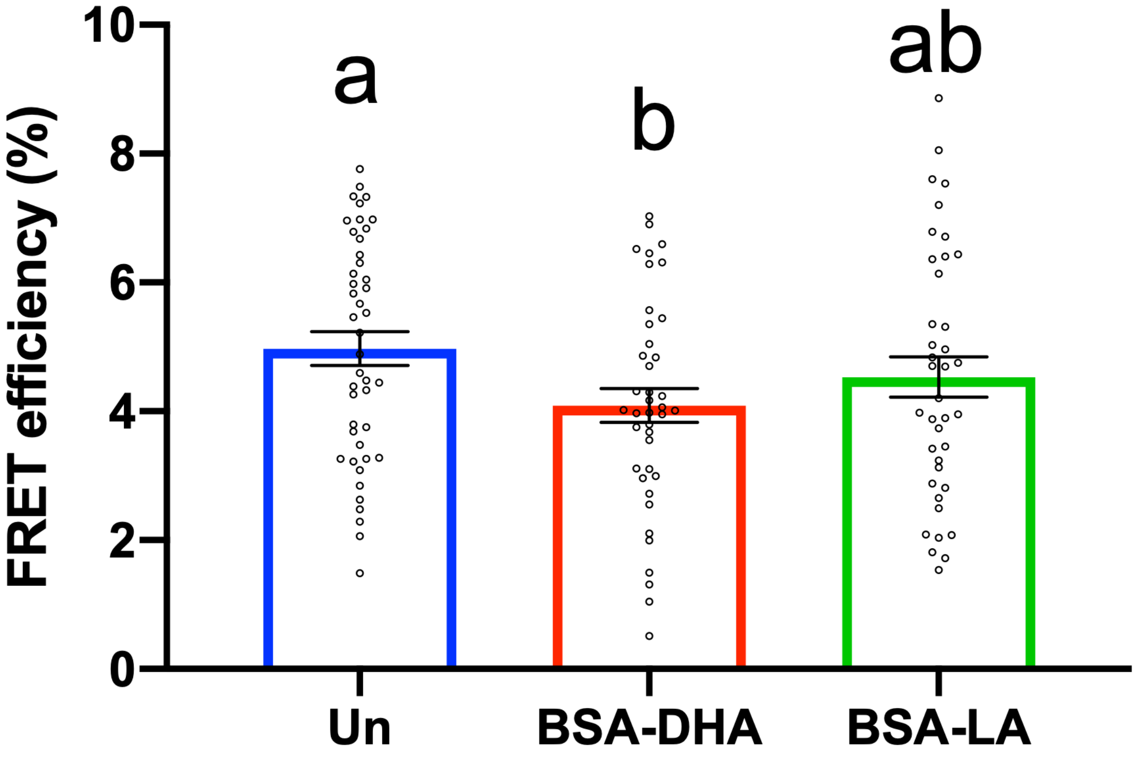

Supplemental Figure S11. DHA reduces EGFR and cholesterol interactions. FLIM-FRET analysis in YAMC cells co-expressing EGFR-GFP and cholesterol sensing D4H-mCherry. YAMC cells were treated with indicated fatty acids (50 μM) for 24 h. Data are presented as mean ± s.e. of the average apparent FRET efficiency per cell. Number of cells examined per treatment, untreated = 44, DHA-BSA = 40 and LA-BSA = 39. Statistical significance between groups as indicated by uncommon letters ($p < 0.05$) was analyzed using one-way ANOVA and uncorrected Fisher's LSD tests.

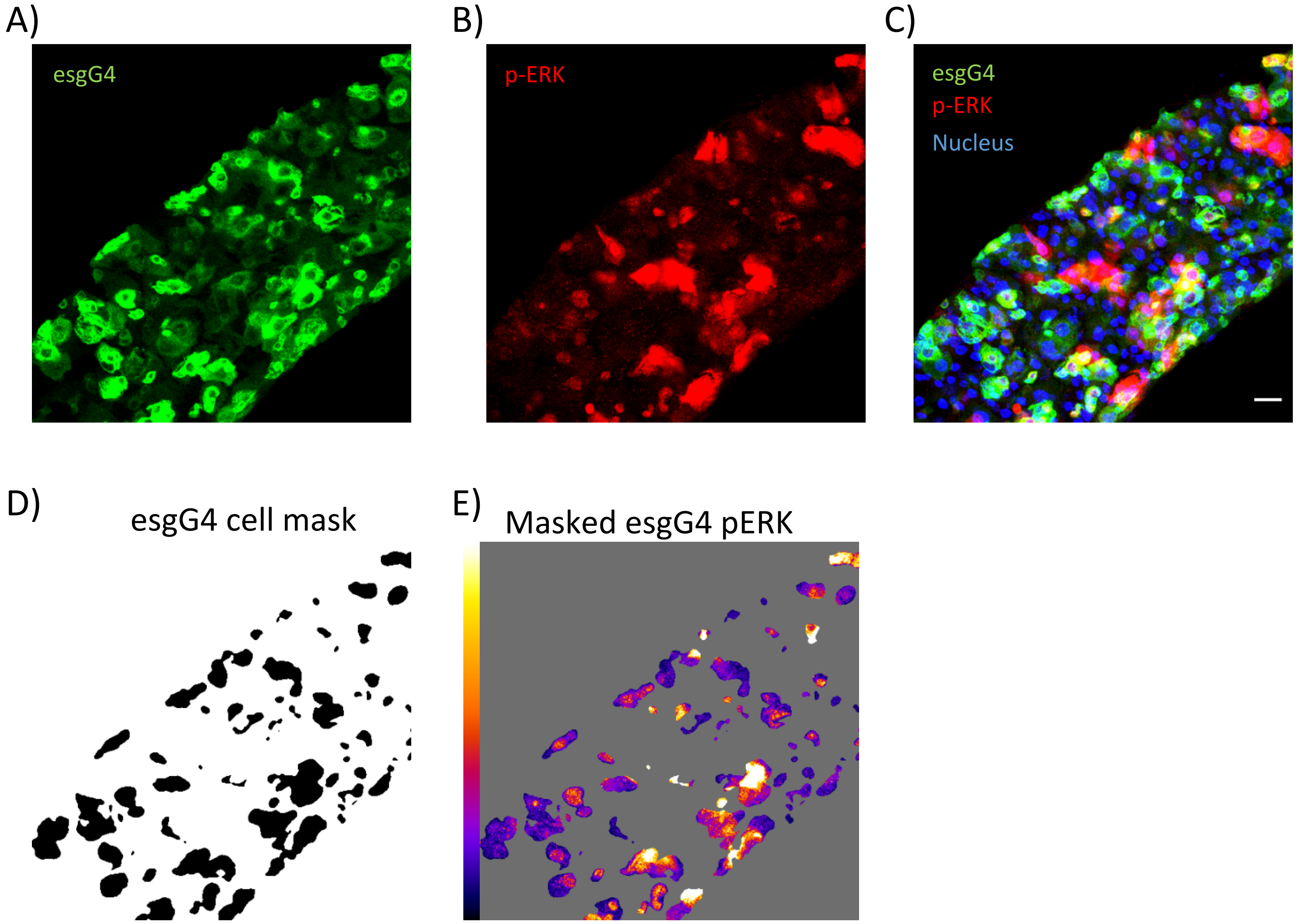

Supplemental Figure S12. Quantitative image-based analysis of pERK in *Drosophila* esgG4 cells. Adult flies were placed on control (PUFA Free), OA or DHA enriched diets for 5 d at 18°C (permissive temperature) before switching to 29°C for 2 d to induce EGFR expression in gut stem cells (GFP). Isolated guts were fixed, permeabilized and labeled with anti-phospho ERK primary antibody (Cell Signaling, #4370), and CF640R (Biotium, Cat# 20178) secondary antibody. Mounted guts were imaged using a Leica confocal microscope using a 40x 1.15 NA oil objective. Approximately 5-10 5 µm sections were acquired to span the gut. Representative maximum image projection of (A) esgG4 cells, (B) pERK and (C) merged images. Scale bar, 20 µm. Representative binary mask identifying (D) esgG4 cells generated from thresholded maximum image projections of esgG4 cells were used as a mask that was then applied to a maximum image projection of (E) pERK.

Supplemental tables:

Supplemental Table S1. Fatty acid profiles of LDL-FA and BSA-FA.

Fatty Acid	LDL-OA	LDL-DHA	BSA-DHA
	(mole%)	(mole%)	(mole%)
14:0	0.47	0.56	1.26
16:0	11.04	11.40	1.79
18:0	5.76	6.67	2.19
18:1n-9 (OA)	68.62		
18:2n-6	6.69	4.86	
20:0	1.70	2.56	2.14
20:3n-3	0.54	0.90	0.79
20:4n-6	1.86	1.02	
22:0	1.99	2.71	2.42
22:6n-3 (DHA)		67.19	87.91

Only selected fatty acids in which at least one observation was > 0.4 mol% are reported. Lipids were extracted from samples and methylated to determine fatty acid profiles by gas chromatography/mass spectrometry.

Supplemental Table S2. Incorporation of exogenous fatty acids into YAMC membrane phospholipids.

Fatty Acid	Untreated	LDL-OA	LDL-DHA	BSA-DHA
	(mole%)	(mole%)	(mole%)	(mole%)
14:0	3.35	0.42	2.41	2.70
16:0	38.00	22.12	37.91	37.19
16:1n-7	4.66	0.33	1.81	2.24
18:0	17.15	12.40	16.40	13.14
18:1n-9 (OA)	21.70	54.15	13.33	13.63
18:1n-7	7.09	1.91	4.53	3.44
18:2n-6	0.77	0.86	2.43	0.88
20:3n-3		1.01	1.08	1.03
20:4n-6	6.58	6.40	2.72	1.49
22:6n-3 (DHA)	0.70	0.41	17.38	24.27

Only selected fatty acids in which at least one observation was > 0.4 mol% are reported. YAMC cells were incubated with select fatty acids (50 μM) for 24 h prior to extraction of total lipids, separation of phospholipids by thin-layer chromatography and determination of fatty acid profile by gas chromatography/mass spectrometry.
